# SIN3B Loss Heats up Cold Tumor Microenvironment to Boost Immunotherapy in Pancreatic Cancer

**DOI:** 10.1002/advs.202402244

**Published:** 2024-09-24

**Authors:** Zhengyan Zhang, Yingying Tang, Yu Wang, Junyi Xu, Xiaotong Yang, Mingzhu Liu, Massimiliano Mazzone, Ningning Niu, Yongwei Sun, Yujie Tang, Jing Xue

**Affiliations:** ^1^ State Key Laboratory of Systems Medicine for Cancer Stem Cell Research Center Ren Ji Hospital Shanghai Cancer Institute Shanghai Jiao Tong University School of Medicine Shanghai 200127 China; ^2^ Department of Oncology Ren Ji Hospital Shanghai Jiao Tong University School of Medicine Shanghai 200127 China; ^3^ Laboratory of Tumor Inflammation and Angiogenesis Center for Cancer Biology VIB Leuven 3000 Belgium; ^4^ Laboratory of Tumor Inflammation and Angiogenesis Center for Cancer Biology Department of Oncology Leuven 3000 Belgium; ^5^ Department of Biliary‐Pancreatic Surgery Ren Ji Hospital Shanghai Jiao Tong University School of Medicine Shanghai 200127 China; ^6^ Key Laboratory of Cell Differentiation and Apoptosis of National Ministry of Education Shanghai Key Laboratory of Reproductive Medicine Department of Histoembryology Genetics and Developmental Biology Shanghai Jiao Tong University School of Medicine Shanghai 200025 China

**Keywords:** anti‐PD1 treatment, CD8+ T cell infiltration, CXCL9/10‐CXCR3 axis, pancreatic ductal adenocarcinoma, tumor microenvironment, Sin3B

## Abstract

Despite progress significant advances in immunotherapy for some solid tumors, pancreatic ductal adenocarcinoma (PDAC) remains unresponsive poorly responsive to such interventions, largely due to its highly immunosuppressive tumor microenvironment (TME) with limited CD8^+^ T cell infiltration. This study explores the role of the epigenetic factor Sin3B in the PDAC TME. Using murine PDAC models, we found that tumor cell‐intrinsic *Sin3B* loss reshapes the TME, increasing CD8^+^ T cell infiltration and cytotoxicity, thus impeding tumor progression and enhancing sensitivity to anti‐PD1 treatment. *Sin3B*‐deficient tumor cells exhibited amplified CXCL9/10 secretion in response to Interferon‐gamma (IFNγ), creating a positive feedback loop via the CXCL9/10‐CXCR3 axis, thereby intensifying the anti‐tumor immune response against PDAC. Mechanistically, extensive epigenetic regulation is uncovered by *Sin3B* loss, particularly enhanced H3K27Ac distribution on genes related to immune responses in PDAC cells. Consistent with the murine model findings, analysis of human PDAC samples revealed a significant inverse correlation between SIN3B levels and both CD8^+^ T cell infiltration and CXCL9/10 expression. Notebly, PDAC patients with lower *SIN3B* expression showed a more favorable response to anti‐PD1 therapy. The findings suggest that targeting SIN3B can enhance cytotoxic T cell infiltration into the tumor site and improve immunotherapy efficacy in PDAC, offering potential avenues for therapeutic biomarker or target in this challenging disease.

## Graphical abstract

*Sin3B* loss reshapes the tumor microenvironment, leading to increased infiltration as well as cytotoxicity of CD8^+^ T cells, to impede PDAC progression and enhance sensitivity to anti‐PD1 treatment. Mechanistically, *Sin3B* deficiency, disrupted SIN3B‐HDAC1/2 complex, thereby altering H3K27Ac distribution, particularly in promoter regions of interferon‐stimulated genes such as *Cxcl10*, enhancing their expression.

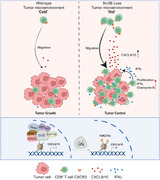

## 1 Introduction

Immunotherapy aimed at impeding immune evasion via the PD‐1 or CTLA‐4 pathways has demonstrated remarkable efficacy for multiple malignancies such as melanoma, non‐small cell lung cancer, and renal cell carcinoma.^[^ ^1^, ^2^ ^]^ Nonetheless, the majority of patients do not respond to immune checkpoint blockade (ICB) therapy. Consequently, understanding the underpinnings of the formation of low‐immune‐response microenvironment and exploring new targets to enhance the sensitivity of tumors to immunotherapy are of paramount importance. Recent studies have highlighted the critical role of intrinsic chromatin regulators within tumor cells in shaping the immune tumor microenvironment (TIME) (e.g., EZH2, SETDB1, and ASF1A).^[^ ^3^, ^4^, ^5^ ^]^ Moreover, the inhibition of epigenetic regulators such as SETDB1, KDM5B, and PHF8 has recently been linked to heightened responsiveness to immunotherapy.^[^ ^6^, ^7^, ^8^ ^]^ These findings suggest that integrating epigenetic therapies could potentially augment the effectiveness of ICB therapy, presenting a promising avenue for cancer treatment.

Recently, Gabriel et al. conducted in vivo epigenetic CRISPR screens using mouse tumor models to systematically identify chromatin regulators that influence tumor immunity and the efficacy of ICB therapies.^[^ ^3^ ^]^ Notably, *Sin3B* emerged as one of the potential candidate genes in this screening. *Sin3B* has been shown to exert dual functions, acting both as activators and repressors of target gene transcription, thereby governing a diverse array of cellular and biological processes. These encompass critical pathways such as cell cycle regulation, senescence, embryonic development, and stem cell differentiation.^[^ ^9^, ^10^, ^11^ ^]^ Earlier investigation has unveiled that *Sin3B* expression is elevated in preneoplastic lesions which is required for oncogenic *Kras*‐induced senescence in vivo.^[^ ^12^ ^]^ However, the role of *Sin3B* in tumor immune evasion remains unexplored.

Pancreatic ductal adenocarcinoma (PDAC) is well‐known for its profoundly immunosuppressive tumor microenvironment (TME), characterized by an abundance of suppressive myeloid cell populations, decreased tumor immunogenicity, and a scarcity of cytotoxic T cell infiltration. These factors collectively contribute to the immunologically “cold” nature of the PDAC TME, presenting a substantial challenge to the success of immunotherapy.^[^ ^13^, ^14^, ^15^ ^]^ In our study, we have unveiled a novel role for *Sin3B* in shaping the immune landscape within tumors, paticular in PDAC. In a murine PDAC model, we found that intrinsic loss of SIN3B within tumor cells enhances CD8^+^ T cell infiltration by increasing the secretion of IFNγ‐induced chemokines CXCL9 and CXCL10. This leads to a positive feedback loop through the CXCL9/10‐CXCR3 axis, transitioning the PDAC microenvironment from “cold” to “hot”. Our findings also reveal that *Sin3B*‐deficient PDAC becomes more responsive to anti‐PD1 therapy. Consistently, analysis of human PDAC samples demonstrates a significant inverse correlation between SIN3B expression and both CD8^+^ T cell infiltration and CXCL9/10 expression. Notably, PDAC patients with lower SIN3B expression show a more favorable response to anti‐PD1 therapy. Mechanistically, *Sin3B* deficiency, disrupted SIN3B‐HDAC1/2 complex, thereby altering H3K27Ac distribution, particularly in promoter regions of interferon‐stimulated genes such as *Cxcl10*, enhancing their expression. Overall, our study identifies SIN3B as a potential therapeutic target for improving cytotoxic T cell accessibility to the tumor microenvironment and enhancing immunotherapy efficacy in the challenging context of PDAC.

## 2 Result

### 2.1 SIN3B Orchestrates Anti‐Tumor Immunity to Regulate In Vivo Pancreatic Tumor Growth

**Figure 1:**
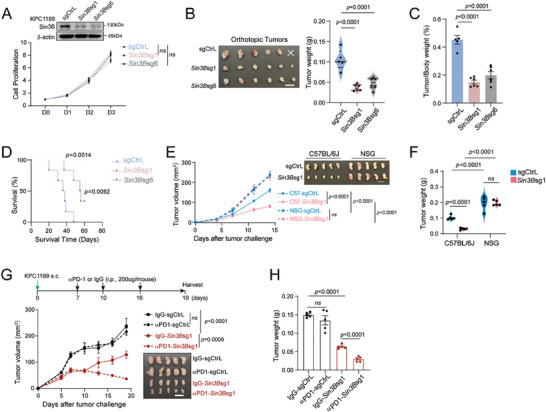
SIN3B orchestrates anti‐tumor immunity to regulate in vivo pancreatic tumor growth. A) The murine pancreatic ductal adenocarcinoma (PDAC) cell line, KPC1199, was subjected to CRISPR‐Cas9‐mediated knockout (KO) of *Sin3B* using control sgRNAs (sgCtrl) and *Sin3B*‐specific sgRNAs (*Sin3B*sg1 and *Sin3B*sg6). In vitro, the proliferation of KPC1199 was assessed through the Luminescent Cell Viability Assay. B‐C) Orthotopic tumor representatives, tumor weight (B), and the tumor/body weight ratio (C) for C57BL/6J wildtype mice inoculated with either sgCtrl or *Sin3B*sg KPC1199 cells (n = 5‐6 per group). D) Survival curves for mice bearing orthotopic tumors and inoculated with either sgCtrl or *Sin3B*sg KPC1199 cells (sgCtrl, n = 6; *Sin3B*sg1, n = 5; *Sin3B*sg6, n = 6). E‐F) Tumor growth curves (E) and weight (F) for NSG mice and C57BL/6J wildtype mice inoculated with sgCtrl or *Sin3B*sg KPC1199 cells (n = 5 per group). G) Schematic of the anti‐PD‐1 treatment schedule(top). Tumor growth was monitored until the experimental endpoints. Tumor growth for isotype (IgG) or a‐PD‐1‐treated C57BL/6J wildtype mice inoculated with sgCtrL or *Sin3B*sg KPC1199 cells (n = 5 for each group) (bottom). H) Subcutaneous tumors from G were harvested at the endpoint, and tumor weights in the groups indicated were shown as a bar graph. For B, E, and G, Scale bar = 1 cm. All data are shown as mean ± SEM. Two‐way ANOVA with multiple comparisons was applied in A, E, and G. One‐way ANOVA with multiple comparisons was applied in B, C, F, and H. The log‐rank test in D. ns, not significant.

To elucidate SIN3B's role in pancreatic ductal adenocarcinoma (PDAC), we employed CRISPR‐Cas9 to specifically knock out (KO) *Sin3B* in the murine PDAC cell line KPC1199, derived from KPC mouse model (*Pdx*‐cre, LSL‐*Kras* ^G12D^, *Trp53* ^R172H^). Our findings revealed that *Sin3B* loss did not impact PDAC cell proliferation in vitro (**Figure** **1**A), but impaired metastatic and invasive capabilities of PDAC cells according to wound healing and transwell‐based migration and invasion assays (Figure S1A–E, Supporting Information). Moreover, in an orthotopic pancreatic cancer mouse model, *Sin3B* deficiency significantly impeded the progression of pancreatic cancer (Figure 1B,C), consequently prolonging the survival of tumor‐bearing mice (Figure 1D).

Based on the differences observed between in vivo and in vitro experiments, as well as previous reported CRISPR screening result, we hypothesized that the inhibitory effect *Sin3B* loss on pancreatic cancer might rely on anti‐tumor immunity. To address this, we inoculated control or *Sin3B*‐deficient KPC1199 cells subcutaneously into the flanks of immunocompetent C57BL/6J and immunodeficient (NOD‐SCID‐*Il2rg*null, NSG) mice, respectively. Remarkably, *Sin3B* deficiency markedly inhibited tumor growth in C57BL/6J mice but only had minimal impact in NSG mice (Figure 1E,F). Given the potential role of *Sin3B* deficiency on anti‐tumor immunity, we further investigated its role in modulating the tumor‐cell response to immunotherapy. In the KPC1199 subcutaneous model, *Sin3B*‐deficient tumors demonstrated enhanced sensitivity to PD‐1 blockade compared to control tumors (Figure 1G,H). These results suggest that *Sin3B* acts as a pivotal regulator in control of anti‐tumor immunity to modulate tumor growth and immunotherapy response in vivo.

### 2.2 SIN3B Loss Reshapes the TIME, Particularly Enhancing CD8^+^ T Cell Infiltration, to Impede Tumor Progression

**Figure 2:**
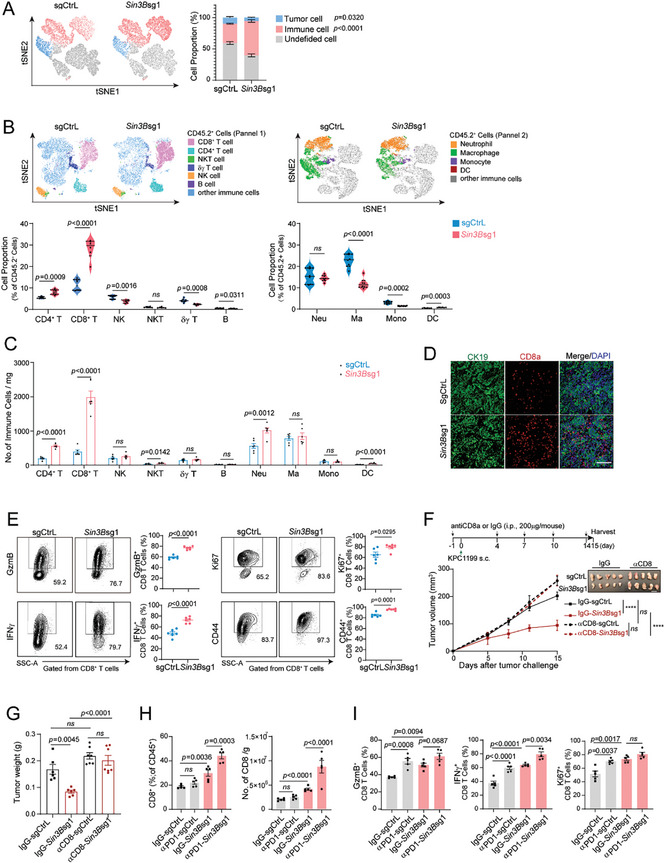
SIN3B loss reshapes the TIME, particularly enhancing CD8^+^ T cell infiltration, to impede tumor progression. A–B) Performance of Barnes‐Hut t‐SNE implementation for flow cytometry data visualization and composition of key immune cells in subcutaneously implanted control and *Sin3B* deficiency tumors (n = 6 per group). Neu, neutrophil. Ma, macrophage. Mono, monocyte. C) Quantification of absolute number of key immune cells infiltrated in control and Sin3B deficiency subcutaneous tumors (n = 6 per group). D) Immunofluorescence staining of tumor cells (CK19) and CD8^+^ T cells (CD8a) in subcutaneous tumors. Scale bar = 40X. E) Representative Contour plots (left) and statistical analysis (right) of Granzyme B (GzmB), IFN𝛾, Ki67, and CD44 production of tumor‐infiltrating CD8^+^ T cells from the indicated mice (n = 6 for each group). F) Schematic of CD8^+^ T cell depletion schedule(top). Tumor growth was monitored until the experimental endpoints. Tumor growth curves for isotype (IgG) or anti‐CD8a C57BL/6J wildtype mice inoculated with either sgCtrL or *Sin3B*sg1 KPC1199 cells (n = 6‐7 for each group) (bottom). Scale bar = 1 cm. G) Subcutaneous tumors from F were harvested at endpoint, and tumor weights in indicated groups were shown as a bar graph. H) Quantification of the ratio (left) and the absolute number (right) of infiltrating CD8^+^ T cells in subcutaneous tumors for IgG‐ or anti‐PD‐1‐treated C57BL/6J wildtype mice inoculated with either sgCtrl or *Sin3B*sg1 KPC1199 cells (n = 5 for each group). I) Statistical analysis of GzmB, IFN𝛾, and Ki67 production of tumor‐infiltrating CD8+ T cells from the IgG‐ or anti‐PD‐1‐treated C57BL/6J wildtype mice. All Data are presented as the mean ± SEM. Unpaired Student's t‐test in B‐E. Two‐way ANOVA with multiple comparisons in F. One‐way ANOVA with multiple comparisons in G‐I. ns, not significant.

Next, we profile the tumor immune microenvironment (TIME) of subcutaneous tumors using flow cytometry and t‐distributed stochastic neighbor embedding (t‐SNE) analysis. We observed a notable shift in immune cell infiltration upon loss of tumor cell‐intrinsic *Sin3B*, with a marked increase in total immune cells (CD45.2^+^) and a decrease in tumor cells (CD45.2^−^; EpCAM^+^) (**Figure** **2**A). Further analysis revealed remarkable alterations in the relative proportion of various immune cell types between the sgCtrL and *Sin3B*sg1 groups (Figure 2B; Figure S2A, Supporting Information). Specifically, the *Sin3B*sg1 group exhibited a substantial increase in both the proportion and absolute number of CD8^+^ T cells within subcutaneous tumors compared to the sgCtrl group, as confirmed by immunofluorescence staining (Figure 2B–D). Additionally, there was a slight increase in CD4^+^ T cell infiltration in the *Sin3B*sg1 group (Figure 2B–C). We also observed a slight decrease in the proportion of other immune cells infiltrating the tumor, such as natural killer cells (NK cells), delta‐gamma T Cells (δγT cells), B lymphocytes (B cells), macrophages, and monocytes; however, their absolute numbers remained relative unchanged (Figure 2B–C). Of note, *Sin3B* loss led to a slight increase in the absolute numbers of NKT, neutrophils, and dendritic cells (DC cells) infiltrating the tumor (Figure 2C). Immune cell types in the spleen did not differ between the two groups (Figure S2B, Supporting Information).

More importantly, *Sin3B* loss enhanced the cytotoxicity and proliferation of CD8^+^ T cells within the tumors, evidenced by increased levels of Granzyme B (GzmB), IFNγ, CD44, and Ki67 in the tumors (Figure 2E). However, there was no significant difference in the expression of CD8^+^ T cell exhaustion markers (PD1, CTLA4, LAG3, and TIM3) between the sgCtrL and *Sin3B*sg1 groups (Figure S2C, Supporting Information). Of note, *Sin3B* loss promoted the Th1 polarization of CD4^+^ T cells as indicated by upregulation of IFNγ (Figure S2D, Supporting Information). Analysis of the phenotype of tumor‐associated macrophages (TAM) and neutrophils suggested no difference in the expression of MHC II, TNFα, and PD‐L1, except for increased expression of iNOS in TAM (Figure S2E–F, Supporting Information). This further supports the notion that the anti‐tumoral function of *Sin3B* loss may rely on substantial infiltration of CD8^+^ T cells. Subsequently, we depleted CD8^+^ T cells in tumor‐bearing mouse, which led to rescue of tumor growth, highlighting the critical role of CD8^+^ T cells in mediating the anti‐tumoral effects of *Sin3B* deficiency (Figure 2F–G; Figure S2H, Supporting Information).

Moreover, administration of α‐PD‐1 further amplified the infiltration of CD8^+^ T cells within the *Sin3B*sg1 group (Figure 2H). This enhanced infiltration was accompanied by an elevated expression of key functional and proliferative markers, including GzmB, IFNγ, and Ki67 (Figure 2I; Figure S2G, Supporting Information). Remarkably, a similar trend was observed in a mouse model of orthotopic pancreatic cancer, where *Sin3B* loss significantly augmented CD8^+^ T cell infiltration, alongside heightened cytotoxicity against tumor cells, as evidenced by the upregulation of IFNγ and GzmB (Figure S3A–C, Supporting Information). These findings robustly demonstrate that *Sin3B* loss reshapes the TIME, particularly through the augmentation of infiltrating CD8^+^ T cells. Taken together, SIN3B modulates tumor progression in a CD8^+^ T cell‐dependent manner.

### 2.3 Minor Contribution of *Col3a1* Downregulation to TIME Remodeling upon *Sin3B* Loss

To uncover the mechanism by which tumor‐intrinsic loss of *Sin3B* heated up cold TME of PDAC, we performed transcriptomic analysis of control and *Sin3B*‐deficienct PDAC cells. Strikingly, the genes downregulated by *Sin3B* loss are mainly associated with pathways such as cell adhesion, extracellular matrix (ECM) organization, collagen trimer, extracellular matrix structural constituent, and other ECM‐related pathways, as revealed by Gene Ontology (GO) and Kyoto Encyclopedia of Genes and Genomes (KEGG) analysis (Figure S4A–B and Table S3, Supporting Information). Delving deeper, in our case, genes involved in the ECM‐related pathways are primarily collagens (Cols) and matrix metalloproteinases (MMPs) (Figure S4C, Supporting Information). Further validation through RT‐PCR confirmed that tumor‐intrinsic loss of *Sin3B* resulted in a decrease in Cols expression in cultured PDAC cells (Figure S4D, Supporting Information), as well as in the orthotopic tumor tissues (Figure S4E, Supporting Information). Through sorting pure tumor cells from the orthotopic tumors, we further confirmed the downregulation of Cols in tumor cells (Figure S4F, Supporting Information).

As widely acknowledged, PDAC is characterized by a dense desmoplastic microenvironment.^[^ ^16^ ^]^ The dense desmoplasia creates a physical barrier to impede the migration of immune cells into the tumor, thereby exacerbating the formation of a “cold” TME in PDAC. Collagens, being the most abundant matrix proteins, are primarily produced by cancer‐associated fibroblasts (CAFs).^[^ ^17^, ^18^ ^]^ However, recent studies highlight the ability of tumor cells to also contribute collagen generation within the tumor stroma. Importantly, collagens derived from tumor cells also play a vital role in shaping the immunosuppressive TME.^[^ ^19^ ^]^ Thus, this promoted us to hypothesize that loss of *Sin3B* might remodel the TIME through the downregulation of collagens. Among the collagens regulated by *Sin3B* loss, *Col3a1* stood out as highly expressed in PDAC tumor cells (Table S3, Supporting Information), thus warranting for further validation.

By employing short hairpin (shRNA) to knockdown (KD) *Col3a1* expression in the KPC1199 cell line, we observed a modest inhibition of PDAC growth (Figure S4G‐H, Supporting Information). Subsequently, profiling the TIME of PDAC revealed that *Col3a1* KD within tumor cells slightly enhanced the infiltration of immune cells, particularly CD8^+^ T cells (Figure S4I,J, Supporting Information). However, no significant changes were observed in the cytotoxicity of CD8^+^ cells (GzmB and IFNγ) or the expression of PD1 (Figure S4K, Supporting Information). Similarly, the levels of IFNγ in CD4^+^ T cells remained unchanged (Figure S4L, Supporting Information). The downregulation of *Col3a1* in *Sin3B*‐deficient PDAC alone cannot fully explain the observed phenotype of TIME remodeling, suggesting that other crucial factors are also at play in shaping the TIME of PDAC in the context of *Sin3B* loss.

### 2.4 SIN3B Loss Enhances T Cell Recruitment in Pancreatic Tumors through CXCL9/10‐CXCR3 Axis

**Figure 3:**
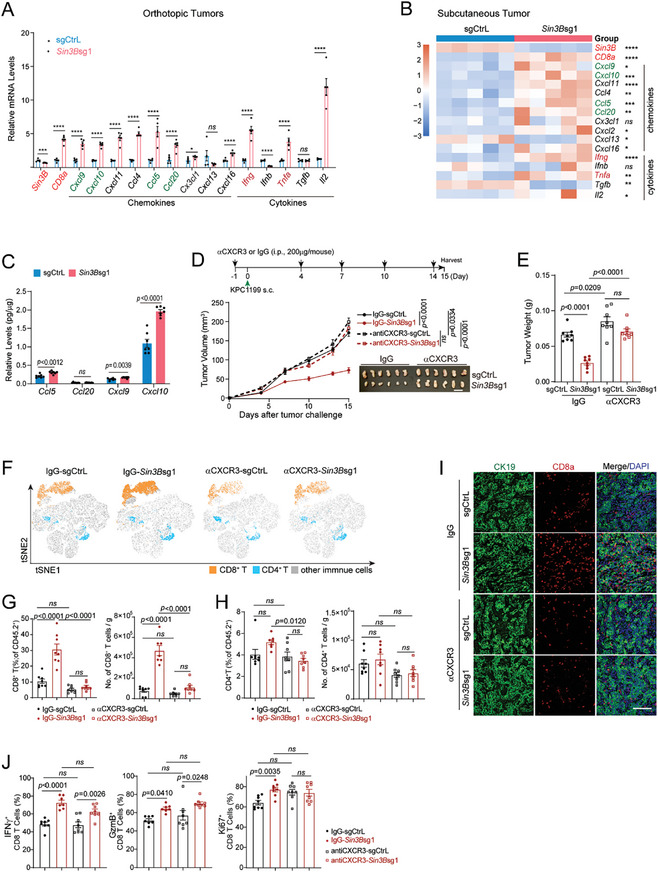
SIN3B loss enhances T cell recruitment in pancreatic tumors through CXCL9/10‐CXCR3 axis. A‐B) The expression levels of selected chemokines and cytokines in orthotopic (A) and subcutaneous (B) tumors were quantified using RT‐PCR (n = 5 for each group). C) Chemokine protein concentrations within subcutaneous tumors were determined using the LEGENDplex Proinflammatory Chemokine Panel. n = 7 for each group. D) Overview of anti‐CXCR3 treatment regimen (top). Tumor growth was tracked to the experimental endpoints. Growth curves of tumors in isotype (IgG) or anti‐CXCR3‐treated C57BL/6J wild‐type mice, inoculated with Control or *Sin3B*‐deficienct KPC1199 cells, are depicted (n = 8 per group) (bottom). E) Bar graph representation of subcutaneous tumor weights harvested at endpoint from the indicated mice. Scale bar = 1 cm. F) t‐SNE plots illustrating the composition of T cells in subcutaneous orthotopic tumors from the indicated mice. G‐H) Quantitative analysis of the ratio (left panel) and absolute count (right panel) of CD8^+^ T cells (G) and CD4^+^ T cells (H) infiltrating subcutaneous tumors in the indicated groups. I) Immunofluorescence staining for tumor cells (CK19) and CD8^+^ T cells (CD8a) in subcutaneous tumor tissues. Scale bar = 40X. J) Statistical analysis of GzmB, IFNγ, and Ki67 production in tumor‐infiltrating CD8^+^ T cells from the indicated mouse groups. All data are presented as the mean ± SEM. The unpaired t‐test was used in A‐C. Two‐way ANOVA with multiple comparisons was applied in D. One‐way ANOVA with multiple comparisons was used in E‐J. ns, not significant, *p < 0.05, **p < 0.01, ***p < 0.001, ****p < 0.0001.

Considering that the infiltration of immune cells into tumor tissues represents an active trafficking process driven by chemo‐attractants, we evaluated the levels of chemokines in both orthotopic and subcutaneous tumors derived from control or *Sin3B*‐deficient PDAC cells. Our finding revealed a significant elevation of various chemokines, including *Cxcl9*, *Cxcl10*, *Cxcl11*, *Ccl5*, and *Ccl20*, which are known for their capability to recruit CD8^+^ T cells.^[^ ^20^ ^]^ Additionally, an increase in cytokines with anti‐tumor properties, including *Ifng*, *Tnfa*, and *Il2*, was also noted (**Figure** **3**A,B). These findings were consistent in both orthotopic and subcutaneous tumor models (Figure 3A,B), further confirming the potent ability of *Sin3B* loss to induce a T cell‐inflamed TME. In our mouse tumor models, functional *Cxcl11*, the third ligand for CXCR3, is lacking in C57BL/6 mice,^[^ ^21^ ^]^ hence subsequent efforts will not focus on *Cxcl11*. Furthermore, we employed the LEGEND plex Proinflammatory Chemokine Panel to measure the protein levels of above chemokines in subcutaneous tumors. While *Sin3B* loss led to a pronounced increase in the mRNA levels of *Ccl5* and *Ccl20*, their protein levels remained relatively low (Figure 3C). In contrast, *Cxcl10* exhibited the highest expression level among all upregulated chemokines (Figure 3C). Given that *Cxcl9* and *Cxcl10* share the same receptor CXCR3,^[^ ^22^ ^]^ we hypothesized that *Sin3B* loss recruited CD8^+^ T cells into the tumor milieu predominantly through the CXCL9/10‐CXCR3 axis.

To this end, wild‐type mice were treated with either anti‐CXCR3 antibodies or isotype controls to block the CXCL9/10‐CXCR3 axis in vivo, as depicted in Figure 3D. Following this intervention, we noted that the CXCR3 blockade led to the abrogation of the anti‐tumor efficacy caused by *Sin3B* deficiency (Figure 3D–E). This was accompanied by a marked reduction in T cell infiltration within the tumors (Figure 3F–H), particularly noting a decrease in CD8^+^ T cells, which was further validated by immunofluorescence staining (Figure 3I). Intriguingly, upon blocking the CXCL9/10‐CXCR3 axis, there was no significant alteration in the cytotoxicity or proliferation of CD8^+^ T cells (Figure 3J). These findings indicate that *Sin3B* loss enhances CD8^+^ T cell infiltration within tumors via the CXCL9/10‐CXCR3 axis, thereby significantly boosting the anti‐tumor immune response.

### 2.5 Tumor Cell‐Intrinsic *Sin3B* Loss Amplifies IFNγ‐Induced CXCL9/10 Secretion in PDAC, Shaping a T Cell‐Inflamed TME

**Figure 4:**
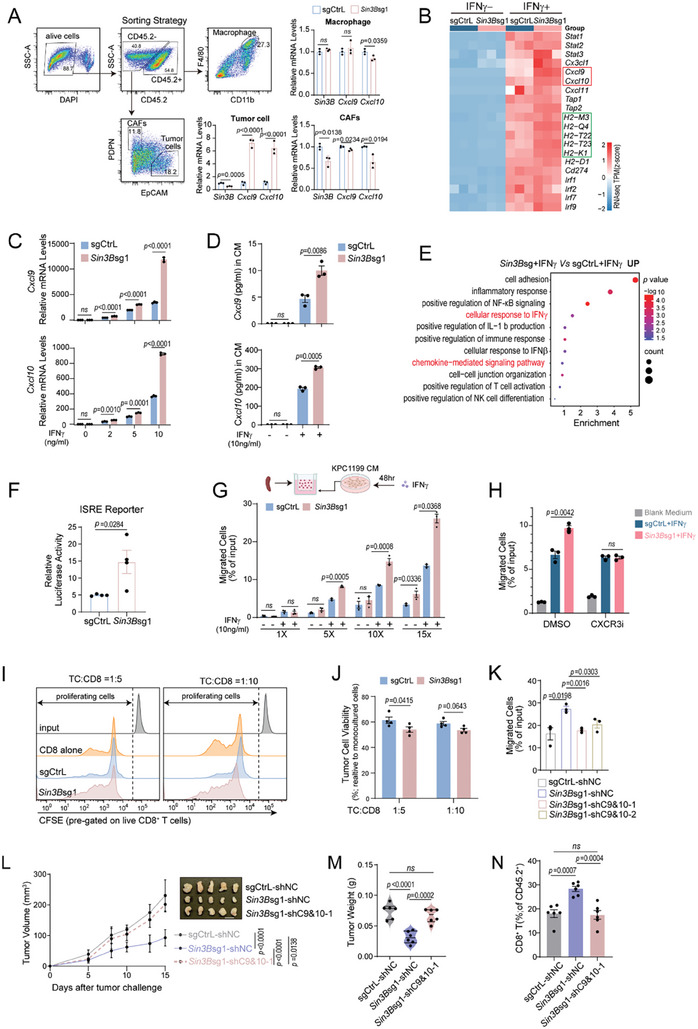
Tumor cell‐intrinsic *Sin3B* loss amplifies IFNγ‐induced CXCL9/10 secretion in PDAC, shaping a T cell–inflamed TME. A)Tumor cells, Cancer‐Associated Fibroblasts (CAFs), and macrophages were isolated from orthotopic tumor tissues via flow cytometry. The plots (left) showed the sorting strategy employed. The bar graph depicts the expression levels of *Cxcl9* and *Cxcl10* in cells sorted specifically, as determined by RT‐PCR. B) A heatmap generated from RNA‐seq displays the expression levels of IFNγ‐induced genes in PDAC cells following 24‐hour treatment with IFNγ (10 ng mL^−1^). C) RT‐PCR analysis quantified *Cxcl9* and *Cxcl10* mRNA expression levels in control and *Sin3B*‐deficienct KPC1199 cells after treatment with varying IFNγ concentrations for 24 h. D) Protein concentrations of *Cxcl9* and *Cxcl10* in conditioned media (CM) from KPC1199 cells treated with IFNγ (10 ng mL^−1^) for 24 h were measured. E) GO enrichment analysis of RNA‐seq data showing enriched pathways in *Sin3B* deficiency compared with Control KPC1199 cells following treatment with IFNγ (10 ng mL^−1^) for 24 h. F) The activity of the IFNγ pathway in control versus *Sin3B*‐deficienct KPC1199 cells was evaluated using a luciferase reporter assay, focusing on the interferon‐stimulated response element (ISRE). G) Schematic diagram and quantification of the CD8^+^ T cell transwell migration assay using KPC1199‐conditioned medium (CM). H) Quantification of the CD8^+^ T cell transwell migration assay using 10X KPC1199‐CM, CD8^+^ T cells were treated with CXCR3 inhibitor (CXCR3i) in vitro. I‐J) Co‐culture of CD8^+^ T cells and control or *Sin3B*‐deficienct KPC1199 cells for 72 h in vitro, tumor cell, CD8^+^ T cell = 1:5 or 1:10. (I) The proliferation of CD8^+^ T cells were measured by CFSE. (J) The viability of tumor cell was measured by Cell‐Titer Glo assay. K) Quantification of the CD8^+^ T cell transwell migration assay using 10X KPC1199‐CM from the indicated groups. NC, negative control. sgCtrL‐shNC, *Sin3B*sg1‐shNC or *Sin3B*sg1‐sh*Cxcl9*&*Cxcl10* (*Sin3*Bsg1‐shC9&10‐1), *Sin3B*sg1‐sh*Cxcl9*&*Cxcl10*‐2 (*Sin3*Bsg1‐shC9&10‐2). L‐M) Tumor growth curves, representatives of tumors (L), and weight (M) for C57BL/6J wildtype mice inoculated with sgCtrL‐shNC, Sin3Bsg1‐shNC or Sin3Bsg1‐shC9&10‐1 KPC1199 cells (n = 6 for each group). Scale bar = 1 cm. N) Quantification of CD8^+^ T cells infiltrated in subcutaneous tumors for indicated mice. All data are presented as the mean ± SEM. Unpaired t‐test in A‐J. One‐way ANOVA with multiple comparisons in K, M and N. Two‐way ANOVA with multiple comparisons in L. ns, not significant.

Previous studies have highlighted macrophages and other stromal cells in the TME as the primary sources of *Cxcl9* and *Cxcl10*.^[^ ^22^ ^]^ Therefore, it is crucial to explore whether the loss of *Sin3B* enhances the secretion of *Cxcl9/10* in stromal cells within the TME or if it amplifies the production of *Cxcl9/10* within the tumor cells themselves. To address this question, we sorted macrophages (CD45.2^+^F4/80^+^), CAFs (CD45.2^−^EpCAM^−^PDPN^+^), and tumor cells (CD45.2^−^PDPN^−^EpCAM^+^) from orthotopic tumors derived from control or *Sin3B*‐deficient groups (**Figure** **4**A). As illustrated, tumor cell‐intrinsic *Sin3B* loss did not enhance the expression levels of *Cxcl9* and *Cxcl10* in either CAFs or macrophages within the TME. However, a notable increase was observed in the tumor cell‐intrinsic expression of *Cxcl9/10* (Figure 4A).

The protein levels of CXCL9 and CXCL10 in conditioned media (CM) derived from PDAC cells in vitro were markedly low, rendering them almost undetectable (Figure S5A, Supporting Information). Notably, CXCL9 and CXCL10 could be typically induced by IFNγ at transcriptional level.^[^ ^22^ ^]^ Consequently, PDAC cells were treated with IFNγ, and further RNA‐seq data unveiled that *Sin3B* loss enhances the expression of *Cxcl9/10*, along with other interferon‐stimulated genes (ISGs), such as antigen‐presenting associated genes (Figure 4B). We further validated that *Sin3B* loss intensified the IFNγ‐induced expression of *Cxcl9/10* both at mRNA and protein levels (Figure 4C–D). Moreover, the expression of *Cxcl9/10* was substantially upregulated in a dosage‐dependent manner in response to IFNγ stimulation (Figure 4C). In addition, pathway analysis showed an enriched expression of genes related to inflammatory response, cellular response to IFNγ, and chemokine‐mediated signaling pathway in *Sin3B*‐deficient PDAC cells (Figure 4E). Consistently, *Sin3B* loss augmented the transcriptional activity of the interferon‐stimulated gene expression based on luciferase reporter assay (Figure 4F). Subsequently, human PDAC cell lines, Mia Paca2 and PANC‐1 were involved. Upon silencing *Sin3B* expression using shRNA in Mia Paca2 and PANC‐1, we observed similar upregulation patterns of *Cxcl*9/10, thereby corroborating our previous findings (Figure S5B–C, Supporting Information).

To delineate whether the recruitment of CD8^+^ T cells rely on chemokines secreted from tumor cells, we conducted an in vitro transwell‐based T cell chemotaxis assay using IFNγ‐treated KPC1199 CM (Figure 4G; Figure S5D, Supporting Information). Our finding revealed that CM from IFNγ‐treated *Sin3B*‐deficient PDAC enhanced CD8^+^ T cell trafficking in a dose‐dependent manner compared to control group (Figure 4G). Furthermore, the administration of a CXCR3 inhibitor markedly attenuated the chemotactic effect of *Sin3B*‐deficient PDAC on CD8^+^ T cells (Figure 4H). Using an in vitro co‐culture system, we demonstrated that *Sin3B*‐deficient PDAC cells could directly enhance the proliferation of CD8^+^ T cells (Figure 4I). Correspondingly, co‐cultured CD8^+^ T cells slightly inhibited the viability of *Sin3B*‐deficient PDAC cells (Figure 4J). However, *Sin3B*‐deficient PDAC cells did not directly impact the cytotoxicity of CD8^+^ T cells in vitro (Figure S5E, Supporting Information).

To functionally assess the involvement of PDAC cell‐derived *Cxcl9/10* in the recruitment of CD8^+^ T cells, we employed shRNA to efficiently and specifically knock down *Cxcl9* and *Cxcl10* together in *Sin3B*‐deficient PDAC cells (termed as *Sin3B*sg1‐ sh C9&10) (Figure S5F, Supporting Information). It was observed that the chemotactic effect on CD8^+^ T cells, instigated by *Sin3B* loss, was attenuated due to silencing of *Cxcl9&10* (Figure 4K). In vivo, tumor growth was rescued by specific knockdown of *Cxcl9&10* in *Sin3B*‐deficient PDAC cells, accompanied by a significant decrease in tumoral infiltrating CD8^+^ T cells (Figure 4L–N). Together, these in vitro and in vivo results demonstrate that *Sin3B* loss enhance the secretion of IFNγ‐induced *Cxcl9/10* derived from PDAC cells, thereby facilitating a T cell‐inflamed TME formation in vivo.

### 2.6 The SIN3B‐HDAC Complex Epigenetically Regulates the Expression of CXCL9/10 through the Re‐Distribution of H3K27Ac

**Figure 5:**
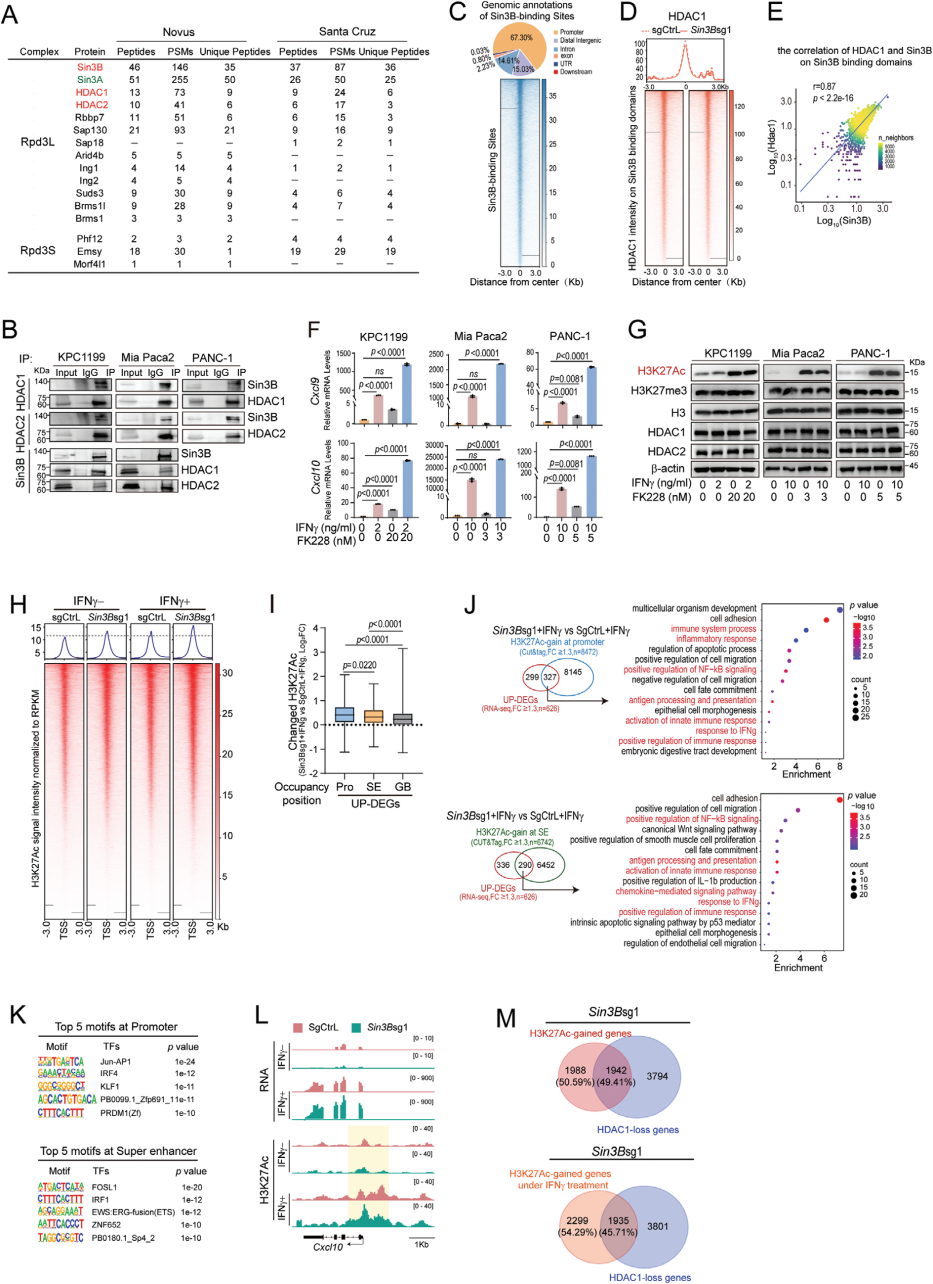
The SIN3B‐HDAC complex epigenetically regulates the expression of CXCL9/10 through the re‐distribution of H3K27Ac. A) Identified proteins were shown in KPC1199 cells through IP‐mass spectrometry using anti‐Sin3B antibodies from Novus and Santa Cruz Biotechnology. PSM, peptide‐spectrum match. B) Co‐immunoprecipitations with anti‐SIN3B, anti‐HDAC1, and anti‐HDAC2 antibody in PDAC cells. Immunoblots were performed using the indicated antibodies. 4% input. IP, immunoprecipitation. C) The distribution of Sin3B‐binding sites in the genome(upper). Heatmap of Sin3B‐binding signals in KPC1199 cells (lower). D) Heatmap of CUT&Tag signals of HDAC1 on Sin3B‐binding domains in control and *Sin3B*‐deficienct KPC1199 cells. The Scale regions were from 3000 bp upstream and downstream of Sin3B‐binding domains. E) the correlation of HDAC1 and Sin3B on Sin3B‐binding domains. F‐G) PDAC cells were treated with the indicated concentrations of FK228 (HDAC1/2 inhibitor). After 24 h, cells were additionally treated with the indicated concentrations of IFNγ for another 24 h. The expression levels of *Cxcl9* and *Cxcl10* in PDAC cells were measured by RT‐PCR(C). Protein levels in PDAC cells were detected by Western blot as indicated(D). H‐L) sgCtrL or *Sin3B*sg1 KPC1199 cells + or – IFNγ indicates that PDAC cells were treated with or without 10 ng mL^−1^ IFNγ for 24 h. H) Heatmap of CUT&Tag signals of H3K27Ac around transcription start sites (TSS) in Control and *Sin3B*‐deficienct KPC1199 cells. The Scale regions were from 3000 bp upstream and downstream of TSS. I) In different regions of the gene, gains of H3K27Ac peak for up‐regulated differentially expressed genes (DEGs) due to *Sin3B* deficiency (based on RNAseq data, *Sin3B*sg1+ IFNγ versus sgCtrL+ IFNγ, Fold change (FC) ≥ 1.3, P value < 0.05). Pro, promoter. SE, super enhancer. GB, gene body. All, Whole gene sequence. J) Venn diagrams depict the overlap of up‐regulated DEGs identified by RNA‐seq (Sin3Bsg1+ IFNγ versus sgCtrL+ IFNγ, FC ≥ 1.3, P value < 0.05) with genes showing H3K27Ac gain (based on Cut&tag RPKM, FC ≥ 1.3) at promoters (upper panel) or super enhancers (SE, lower panel) in *Sin3B*‐deficienct KPC1199 cells. The overlapping genes were subjected to GO functional enrichment analysis. K)Additionally, top‐scoring transcription factor (TF) motifs identified by HOMER de novo motif analyses were based on these overlapping genes(H). L) Integrative Genomics Viewer (IGV) plots showed the RNA‐seq peaks (upper panel) and CUT&Tag peaks of H3K27Ac (lower panel) at the *Cxcl10* sites in control and *Sin3B*‐deficienct KPC1199 cells. M) The Venn diagram indicates the overlap between H3K27Ac‐gained genes and HDAC1‐loss genes in Sin3B‐deficient KPC1199 cells (upper). And the overlap between H3K27Ac‐gained genes following IFNγ treatment and HDAC1‐loss genes in Sin3B‐deficient KPC1199 cells (lower). One‐way ANOVA with multiple comparisons in F.

SIN3B lacking identifiable DNA‐binding motifs and enzymatic activities, serves as a molecular scaffold that orchestrates transcriptional regulation through the formation of a complex via protein‐protein interactions.^[^ ^10^ ^]^ We conducted IP‐mass spectrometry on KPC1199 cells using two SIN3B antibodies from Novus and Santa Cruz Biotechnology, respectively. Consistent with previous reports,^[^ ^10^, ^23^, ^24^ ^]^ we identified several proteins from canonical SIN3‐HDAC (large SIN3 complex, Rpd3L) and smaller SIN3B (Rpd3S) complexes (**Figure** **5**A). Interaction confirmation between Sin3B and HDAC1/2 was achieved via immunoprecipitation across murine and human PDAC cell lines (Figure 5B). Thus, we further explore whether SINB exerted its function through SIN3B‐HDAC complex. We first performed co‐immunoprecipitation experiments targeting HDAC1, which demonstrated that *Sin3B* loss significantly weakened the interaction between RBBP7 and HDAC1 (Figure S6A, Supporting Information). Subsequently, we carried out CUT&Tag assay for Sin3B and HDAC1, defining a 6kb‐sized Sin3B‐binding domain based on unique peaks in control versus *Sin3B*‐deficient PDAC cells (Figure 5C). Most importantly, *Sin3B* loss resulted in diminished HDAC1 signals at these SIN3B‐binding domains (Figure 5D). Notably, within the Sin3B‐binding domain, the signal intensity of Sin3B exhibited a strong positive correlation with that of HDAC1 (r = 0.87; *p* < 2.2e‐16) (Figure 5E). PDAC cells treated with the HDAC1/2‐specific inhibitor FK228 intensified the upregulation of *Cxcl9/10* in response to IFNγ stimulation (Figure 5F), which was consistent with the result after *Sin3B* loss in PDAC cells (Figure 4). These findings demonstrate that *Sin3B* loss disrupts the Sin3B‐HDAC complex, resulting in reduced HDAC1 occupancy at the Sin3B domain.

As expected, FK228 treatment increased H3K27Ac protein levels (Figure 5G), suggesting the SIN3B‐HDAC complex may primarily exert its epigenetic regulation by modulating H3K27Ac. *Sin3B* loss didn't significantly alter total H3K27Ac levels, as well as other histone marks (Figure S6B, Supporting Information). However, *Sin3B* loss led to H3K27Ac deposition across the genome regardless of IFNγ conditions (Figure S6C, Supporting Information). Additionally, H3K27Ac signals, primarily enriched at gene promoters, were elevated in *Sin3B*‐deficient PDAC with or without IFNγ stimulation (Figure 5H). Importantly, upregulated genes caused by *Sin3B* loss were closely associated with H3K27Ac signal presence in regions of gene promoter and super‐enhancer, compared to gene body (Figure 5I). This prompts the question of whether upregulated‐genes due to H3K27Ac‐redistribition, modulated by the SIN3B‐HDAC complex, are involved in immune regulation. To address this issue, we integrative analysis of differentially expressed genes (DEGs, n = 626) that were up‐regulated due to *Sin3B* loss, together with genes exhibiting enriched H3K27Ac signals at promoter regions (n = 8472) or super‐enhancer (SE, n = 6742) regions upon *Sin3B* loss (Table S4, Supporting Information; Figure 5J). Utilizing GO functional enrichment analysis, we identified that the overlapping genes exhibit a significant association with positive regulation of immune response, inflammatory processes, antigen processing and presentation, chemokine‐mediated signaling pathway, and response to IFNγ (Figure 5J). We also observed that genes upregulated due to the *Sin3B* loss are highly associated with cell fate commitment, consistent with previous functional studies of Sin3B^[^ ^10^ ^]^ (Figure 5J). Moreover, top‐scoring transcription factor (TF) motifs were identified within these overlapping genes using HOMER de novo motif analysis. Additionally, we observed an enrichment of H3K27Ac peaks at the binding sites for IRF4, IRF1, and PRDM1 (Zf), which were highly associated with IFNγ response (Figure 5K). In more detail, *Sin3B* loss resulted in an enrichment of H3K27Ac peaks in the promoter regions of *Cxcl10* (Figure 5L) and at super enhancer of *Cxcl9* (Table S4, Supporting Information) in KPC1199 cells. Furthermore, the H3K27Ac peaks on *H2‐T23*, *H2‐T22*, *H2‐Q4*, *Ifi206*, and *Mndal* were significantly increased upon *Sin3B* deficiency (Figure S6D, Supporting Information). Next, we determined whether the upregulated H3K27Ac was directly dependent on HDAC loss. Upon the loss of *Sin3B*, ≈49.41% of genes with increased H3K27Ac levels overlapped with genes showing reduced HDAC1 signal. Similar patterns were observed in H3K27Ac‐gained genes further induced by IFNγ (Figure 5M). Interestingly, only half of the observed increase in H3K27Ac deposition can be attributed directly to HDAC1 loss, suggesting that *Sin3B* loss affects H3K27Ac enrichment through both HDAC1‐direct and indirect mechanisms. A recent study^[^ ^25^ ^]^ revealed that HDACs inhibit SWI/SNF subfamily remodelers, which are involved in chromatin unraveling, thereby stabilizing modified nucleosomes that preserve gene silencing. This mechanism may explain the global upregulation of H3K27Ac.

Protein mass spectrometry (Figure 5A) revealed the presence of Sin3A alongside Sin3B. Given Sin3A and Sin3B share high sequence similarity, whether Sin3A has similar impact on the tumor microenvironment was evaluated. As illustrated, *Sin3A* loss didn't affect tumor growth, as well as infiltrating immune cells such as CD8^+^ T cells (Figure S7A–B, Supporting Information). What's more, the mRNA levels of *Cxcl9*, *Ifng*, and *Tnfa* were decreased (Figure S7C, Supporting Information). These results highlight distinct roles of SIN3A and SIN3B in tumor immune microenvironment, and only *Sin3B* loss triggering an anti‐tumor response in PDAC.

### 2.7 SIN3B Loss Enhances Immune Response and Sensitivity to Immunotherapy in Human Patients with PDAC

**Figure 6:**
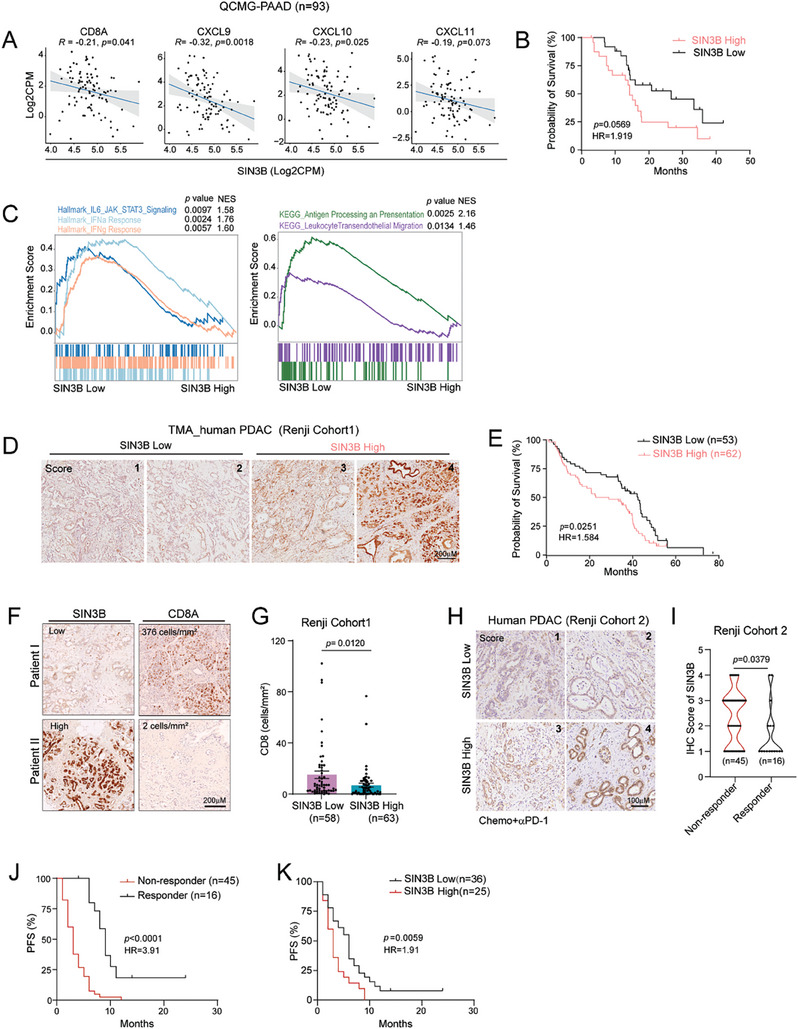
SIN3B loss enhances immune response and sensitivity to immunotherapy in patients with PDAC. A‐C) Analysis of human PDAC Cohort (QCMG, Nature 2016). (A) Pearson's correlation analysis plots comparing expression of *SIN3B* with the expression of *CD8a*, *CXCL9*, *CXCL10* or *CXCL11* in human primary PDAC transcriptomic data from QCMG‐Data (n = 93 samples). The line represents the line of best fit. B) Kaplan–Meier survival curve of human PDAC patients stratified based on SIN3B expression levels. Statistics by log‐rank test (*SIN3B* Low, 25% Bottom, n = 25; *SIN3B* High, 25% Top, n = 25). (C) GSEA was performed comparing lower quartile *SIN3B* expressers (*SIN3B* Low; 25% Bottom) with the upper quartile expressers (*Sin3B* High; 25% Top) within the human PDAC cohort. D‐G) Human PDAC corhort1 from Renji Hospital. (D) Representative SIN3B intensity score of IHC staining in human PDAC tissues. Scale bars, 200 µm. (E) Kaplan–Meier survival curve of human PDAC patients stratified based on SIN3B expression levels. Statistics by log‐rank test (SIN3B Low, n = 53; SIN3B High, n = 62). (F) Representative IHC staining of SIN3B and CD8A in human PDAC tumor (Renji cohort 1). Scar bar, 200um. (G) Quantitative analysis of CD8 intensity (cells mm^−2^) in tumor‐adjacent tissues from two groups of PDAC patients with the low and high SIN3B levels. H‐K) The analysis of human PDAC corhort2 prior to chemotherapy and anti‐PD1therapy from Renji Hospital. (H) Representative images of IHC staining of SIN3B in human PDAC. Scar bar, 100 µM. (I) IHC score of SIN3B in responders and non‐responders to anti‐PD‐1 therapy. (J) Comparison of Progression‐Free Survival (PFS) of PDAC patients treated with anti‐PD1 in non‐responder (n = 45) and responder (n = 16) groups. Statistics by log‐rank test. (K) Comparison of PFS of PDAC patients treated with anti‐PD1 in SIN3B High (n = 25) and SIN3B Low (n = 36) groups. Statistics by log‐rank test.

Given that *Sin3B* loss triggered a T‐cell inflamed TME and significantly enhanced sensitivity to anti‐PD‐1 therapy in mouse models of PDAC. We also observed that *Sin3B* loss similarly enhanced the IFNγ‐induced expression of *Cxcl*9/10 in human PDAC cell lines (Figure S5C, Supporting Information). Thus, we analyzed data from a human PDAC database (QCMG) comprising 93 samples, revealing a negative correlation between *SIN3B* and *CD8A* levels in PDAC tumors (**Figure** **6**A). Moreover, *SIN3B* expression inversely correlated with chemokines (*CXCL9*, *CXCL10*, and *CXCL11*) known to attract CD8^+^ T cells, and high *SIN3B* levels were linked to poor prognosis in PDAC patients (Figure 6A,B). Gene Set Enrichment Analysis (GSEA) revealed that PDAC patients with low *SIN3B* levels exhibited upregulation of immune‐related pathways, including IFNγ response, IFNα response, IL6 JAK/STAT3 signaling, leukocyte migration, and antigen processing and presentation (Figure 6C). Similarly, analysis of another public dataset containing 117 PDAC patient samples (GSE62165),^[^ ^26^ ^]^ further confirmed the negative correlation between *SIN3B* expression and the levels of IFNγ‐induced chemokines (*CXCL9*, *CXCL10*, and *CXCL11*) and cytokines (*IFNG* and *GZMB*) (Figure S8A, Supporting Information). PDAC with low *SIN3B* levels also demonstrated upregulated pathways related to inflammatory response and IFNα response (Figure S8B–C, Supporting Information). In database of CPTAC (n = 140), we also observed a significant negative correlation between *SIN3B* and *CXCL9/10/11* (Figure S8D, Supporting Information).

To assess potential differences in *SIN3B* expression and functionality between these subtypes, we utilized four human PDAC transcriptomics databases (QCMG, CPTAC, TCGA, and GSE62165) and classified PDAC into basal‐like and classical subtypes^[^ ^27^ ^]^ using NMF clustering analysis (Figure S9A‐B, Supporting Information). In the CPTAC, TCGA, and GSE62165 datasets, no significant differences in SIN3B expression were observed between the two subtypes. However, a slight increase in SIN3B levels was noted in the basal‐like subtype within the QCMG dataset (Figure S9C, Supporting Information). Our research suggests that *SIN3B* expression is inversely correlated with CD8A and its chemokines *CXCL9/10/11* levels in the QCMG, CPTAC, and GSE62165 datasets, excluding the TCGA database. Across basal‐like and classical subtypes, despite some fluctuations in the correlation coefficients (R values), the trend remains consistent (Figure S9D, Supporting Information). These results indicate that the immune modulatory function of SIN3B is independent of the PDAC subtype.

In a cohort of surgical PDAC tumor samples from Renji Hospital (Renji Cohort 1), high SIN3B protein levels were associated with poor prognosis by Kaplan‐Meier survival analysis (Figure 6D,E). Tumors with low SIN3B expression exhibited greater infiltration of CD8^+^ T cells in paired peri‐carcinomatous tissues (Figure 6F,G), suggesting a potential correlation with ICB therapy response. To this end, a retrospective study involving 61 advanced PDAC patients who received chemotherapy combined with anti‐PD1 treatment was included (Renji Cohort 2). Our finding revealed that patients responding positively to anti‐PD1 therapy had reduced SIN3B protein levels, correlating with prolonged progression‐free survival (PFS) (Figure 6H–J). Moreover, patients with lower SIN3B protein levels demonstrated significantly prolonged PFS following anti‐PD‐1 treatment (Figure 6K). Together, these results indicate that SIN3B levels are inversely correlated with anti‐tumor immune response and ICB therapy response in PDAC, suggesting its potential as a predictive biomarker for immunotherapy outcomes in PDAC patients.

## 3 Discussion

Epigenetic regulators have recently been implicated in immune escape and sensitivity to immunotherapy, presenting an appealing approach for directly targeting epigenetic factors or combining epigenetic therapies with immunotherapy in PDAC.^[^ ^4^, ^8^, ^28^, ^29^ ^]^ CRISPR loss‐of‐function screens have provided a way to identify novel epigenetic regulators of anti‐tumor immunity. Of note, a study from Gabriel group conducted in vivo epigenetic CRISPR screens using mouse tumor models to systematically identify chromatin regulators influencing tumor immunity and the efficacy of ICB therapies,^[^ ^3^ ^]^ SIN3B emerged as one of the potential negative candidates of anti‐tumor immunity. However, the role of SIN3B in tumor immunity hasn't been defined yet. Our study is the first to unveil the regulatory role of SIN3B in the tumor immune microenvironment. In murine PDCA models, tumor cell‐intrinsic *Sin3B* loss reshapes the TIME, characterized by a notable influx of immune cells into the tumor tissue, particularly an augmented presence and activity of tumor‐infiltrating CD8^+^ T cells. Notably, our analysis of human PDAC tumor tissues revealed a consistent pattern, with lower levels of SIN3B associated with increased infiltration of CD8^+^ T cells. These findings suggest a conserved role of SIN3B in shaping the TIME across species. Moreover, we observed that *Sin3B* loss enhances the susceptibility of PDAC in mice to anti‐PD1 therapy. In consistent, analysis of PDAC patients who exhibited a favorable response to ICB therapy revealed lower expression levels of SIN3B in tumor tissues compared to non‐responders.

Our research further revealed that the infiltration of effector CD8^+^ T cells in PDAC is dependent on the CXCL9/10‐CXCR3 axis. *Sin3B* deficiency results in the upregulation of chemokines *Cxcl9/10*, thereby enabling the high levels of *Cxcl9/10* in the tumor tissues to recruit more CXCR3‐expressing effector T cells. These activated effector T cells will secrete a larger amount of IFNγ, which activates the JAK‐STAT signaling pathway within PDAC cells, further inducing the expression of *Cxcl9/10*, and other ISGs such as antigen‐presenting associated genes. Consequently, the recruitment of effector CD8^+^ T cells and *Sin3B*‐deficient PDAC cells form a positive feedback loop, amplifying the anti‐tumor effects and transforming the PDAC from a “cold” tumor to a “hot” tumor. Regarding whether the expression of *Cxcl9/10* is subject to epigenetic regulation, our studies found that SIN3Bplays an epigenetic regulatory role through the SIN3B‐HDAC complex. It was demonstrated that *Sin3B* loss results in an increased enrichment of H3K27Ac peaks at the promoter or super enhancer regions of *Cxcl9*, *Cxcl10*, *H2‐T23*, *H2‐T22*, *H2‐Q4*, and other ISGs. CXCR3‐expressed T cells homing to tumor site play a crucial role in anti‐tumor immunity and anti‐PD‐1 treatment efficacy.^[^ ^30^, ^31^ ^]^ Therefore, our research provides robust evidence for targeting epigenetic therapy to modulate anti‐tumor immune responses and enhance immunotherapy.

Previous studies have demonstrated that levels of SIN3B are upregulated in pancreatic intraepithelial neoplasia (PanIN) in both humans and mice.^[^ ^32^ ^]^ Research has revealed that the deletion of *Sin3B* in the mouse pancreas can delay the progression of pancreatic tumors.^[^ ^33^ ^]^ This effect is primarily attributed to the role of SIN3B in mediating RAS‐induced cellular senescence.^[^ ^32^, ^33^, ^34^ ^]^ In the context of cancer, cellular senescence was initially considered to act as a barrier to tumor progression, such as prostate cancer.^[^ ^35^ ^]^ However, it is well‐established that senescent cells secrete a set of inflammatory cytokines, collectively referred to as the SASP (senescence‐associated secretory phenotype).^[^ ^36^ ^]^ Given the significant role of inflammation in the progression of pancreatic cancer, the deletion of *Sin3B* in the pancreas prevents oncogenic *Kras*‐induced senescence, correlating with a reduction in the proinflammatory phenotype, ultimately resulting in delayed pancreatic tumor progression.^[^ ^33^ ^]^ Surprisingly, our research indicates that at the stage of PDAC, SIN3B participates in and promotes the progression of PDAC in a different manner. As described above, SIN3B contributes to the pancreatic TIME remodeling at the epigenetic level by regulating the expression of chemokines for CD8^+^ T cells. The intrinsic loss of *Sin3B* in PDAC cells promotes the formation of a T cell‐inflamed TME. This suggests that SIN3B, as an epigenetic regulator, plays distinct roles in different tumors or even at different stages of the same type of tumor. Therefore, our study provides a novel perspective and insight into the role of SIN3B in pancreatic tumor development.

In mammals, the SIN3 family is comprised of two isoforms, namely SIN3A and SIN3B, which are encoded by homologous genes and exhibit broadly overlapping expression patterns in their proteins. Both Sin3A and Sin3B harbor four paired amphipathic α‐helices (PAH) known as PAH domains, a central HDAC interaction domain (HID) to which almost all core corepressor components bind, and a C‐terminal highly conserved region (HCR).^[^ ^10^, ^23^, ^34^, ^37^ ^]^ Through these domains, SIN3A/B engages in interactions with partner proteins, notably HDAC1/2, thereby forming the SIN3A‐HDAC and Sin3B‐HDAC complexes, which perform biological functions.^[^ ^23^ ^]^ However, findings indicate that the *Sin3A* deficiency in PDAC cells does not result in an increased infiltration of CD8^+^ T cells, but rather exhibits a declining trend. Moreover, *Sin3A* loss leads to decreased expression of *Cxcl9*, *Ifng*, and *Tnfa* in pancreatic tumor tissues, suggesting that SIN3A plays a distinct role in the regulation of the TIME, differing from that of SIN3B. This also opens up the possibility of SIN3B as a precise epigenetic target for therapeutic intervention.

Despite the current approval of HDAC enzyme activity inhibitors such as Vorinostat (SAHA), Belinostat, Panobinostat, and Romidepsin for the treatment of diseases like myeloma and lymphoma, these HDAC inhibitors have demonstrated limited efficacy in solid tumors and are associated with certain side effects. This is primarily due to the challenge in developing inhibitors that are specific to each HDAC isoforms.^[^ ^38^, ^39^, ^40^, ^41^, ^42^ ^]^ Additionally, HDACs are typically part of various complexes; for example, HDAC1/2 not only forms the SIN3‐HDAC complex with SIN3A/B but also forms the NuRD‐HDAC and CoREST‐HDAC complexes with CHD3/4 and CoREST, respectively. These different complexes play varied biological roles in cellular processes, posing significant challenges in clinical treatment by targeting HDAC enzyme activity alone.^[^ ^42^, ^43^, ^44^, ^45^, ^46^, ^47^ ^]^ Therefore, understanding the roles of different HDAC complexes in tumors and further identifying specific targets for these complexes are crucial. Notably, the Samuel Waxman team designed and constructed a peptide that specifically binds to the SIN3 PAH2 domain, acting as a competitive binding decoy.^[^ ^48^, ^49^ ^]^ This development greatly enhances the potential of targeting SIN3B as a clinical therapeutic target. However, we must also consider the possible side effects that may arise when designing inhibitors or decoys aimed at disrupting the SIN3‐HDAC complex to target SIN3B as a therapeutic intervention.

Under normal physiological conditions, the SIN3B‐HDAC complex is involved in the regulation of embryonic development, cell differentiation, and the cell cycle regulation.^[^ ^9^, ^34^, ^50^ ^]^ Therefore, targeting SIN3B to disrupt this complex can also impair its function in normal human cells, leading to cytotoxicity. Additionally, the protein sequences and structures of SIN3A and SIN3B are highly similar, with both interacting with HDAC1/2 through the central HDAC interaction domain to form the SIN3A‐HDAC and SIN3B‐HDAC complexes,^[^ ^11^ ^]^ which perform distinct biological functions. Despite our findings that Sin3A deficiency in PDAC cells does not lead to increased infiltration of CD8^+^ T cells, SIN3A also plays a crucial and extensive regulatory role. For instance, in the development of immune cells, deletion of *Sin3A* affects thymic cellularity and the count of CD8+ T cells.^[^ ^51^ ^]^ The SIN3A‐SIN3HDAC corepressor complex maintains embryonic stem cell (ESC) pluripotency and promotes the generation of induced pluripotent stem cells (iPSCs).^[^ ^52^ ^]^ Due to the highly similar sequences of SIN3A and SIN3B, inhibitors targeting SIN3B might inadvertently target SIN3A, thereby causing side effects. Consequently, when targeting SIN3B to disrupt the SIN3B‐HDAC complex, it is essential to consider both the safe and effective concentration of SIN3B inhibitors or decoys and to explore binding sites with protein sequences or domains that are distinct from those of SIN3A, in order to maximize the specificity of SIN3B inhibitors.

To explore the potential of SIN3B as a therapeutic target in other cancers, we analyzed transcriptomic data from human colon cancer, lung cancer, and breast cancer obtained from the CPTAC database. In the human colorectal cancer (CRC) cohort, *SIN3B* expression levels were significantly negatively correlated with *CXCL9/10/11* as well as *CD8A, GzmA*, and *GzmB* (Figure S10A, Supporting Information). This finding suggests that colon tumor tissues with low SIN3B expression may exhibit greater CD8^+^ T cell infiltration. Further GSEA revealed significant enrichment of IFNα and IFNγ response pathways, chemokine signaling, and cytokine receptor interactions in the low SIN3B expression group (Figure S10B,C, Supporting Information). Conversely, in the lung cancer and breast cancer cohorts, *SIN3B* did not exhibit any correlation with *CXCL9/10/11* or *CD8A* (Figure S10D,E, Supporting Information). These results indicate that SIN3B may have a role in regulating the immune microenvironment and suggest that targeting SIN3B could potentially enhance immunotherapy efficacy in colon cancer.

In summary, our findings unveil the novel role of SIN3B in mediating immune escape in PDAC tumors. The loss of *Sin3B* reshapes the TIME through the CXCL9/10‐CXCR3 axis, particularly boosting the infiltration of CD8^+^ T cells and enhancing the response to anti‐PD‐1 therapy. In the tumor microenvironment of PDAC, *Sin3B*‐deficient tumor cells and the recruitment of CD8^+^ T cells establish a positive feedback loop mediated by IFNγ‐CXCL9/10. Furthermore, the loss of *Sin3B* directly influences the redistribution of H3K27Ac, serving as a widespread and pivotal regulatory mechanism in the epigenetic landscape. This ultimately promotes the formation of a T‐cell inflamed tumor microenvironment. Collectively, our findings propose a potential target for enhancing the accessibility of cytotoxic T cells to the tumor site and improving immunotherapy of PDAC.

## 4 Experimental Section

### Cell Lines

Murine PDAC cell line, KPC1199, was generated from spontaneous pancreatic tumors in *Pdx^cre^*; *LSL‐Kras^G12D^; Trp53^R172H^* genetic mouse model (KPC) as previously described. Mia Paca2 and PANC‐1 and 293T were purchased from the American Type Culture Collection (ATCC). All cells were maintained in a humidified incubator at 37 °C with 5% CO_2_ and grown in Dulbecco's modified Eagle's medium (DMEM) supplemented with 10% fetal bovine serum (FBS) and 100 IU ml^−1^ of penicillin/streptomycin (P/S). All cell lines used were tested negative for Mycoplasma.

### Animal Experiments and Tumor Challenges

All mouse experiments were approved by the Animal Care and Use Committees at Ren Ji Hospital (RJ2020‐0505), Shanghai Jiao Tong University School of Medicine. All mice used in our experiments were male and between six and eight weeks of age, which were housed under standard Specific pathogen‐free conditions. C57BL/6J wild‐type mice were purchased from Shanghai SLAC Laboratory Animal Co., Ltd. (Shanghai, China). NSG (NOD‐SCID‐*Il2rg^null^*) mice were obtained from Shanghai Model Organisms Center, Inc. (Shanghai, China). For subcutaneous tumor model, 1 × 10^6^ KPC1199 cells were resuspended in 100 µL of Matrigel (Corning,354 234), HBSS (1:9) solution and subcutaneously injected on day 0. For orthotopic tumor model, 1 × 10^6^ KPC1199 cells in 25 µL of Matrigel, HBSS (1:9) solution were injected in pancreas.

*For CD8^+^ T cell depletion studies*, 200 µg of rat anti‐CD8a (Bio X Cell, clone 53–6.7, BE0004‐1) or rat IgG2a isotype control (Bio X Cell, clone 2A3, BE0089) were injected intraperitoneally starting on day −1 before tumor challenge, and administrated twice a week, until day 15.

In vivo *CXCR3 neutralization*, mice were injected intraperitoneally with anti‐CXCR3 (Bio X Cell; clone CXCR3‐173; BE0249) or matched Armenian hamster IgG isotype control (Bio X Cell, Clone, Polyclonal, BE0091) at a dose of 200 µg per mouse on 1 day prior to tumor inoculation, and administrated twice a week after tumor challenge, until day 15.

*For anti‐PD‐1 immunotherapy experiments*, mice received intraperitoneal injection of 200 µg anti‐mouse PD‐1 antibody (Bio X Cell, clone RMP1‐14, BE0146) or matched 200 µg rat IgG2a isotype control (Bio X Cell, clone 2A3, BE0089) every 3–4 days from day 7 after tumor challenge until indicated time points.

The tumor size was monitored and measured every 3–4 days by vernier caliper to collect maximal tumor length and width. Tumor volume was calculated according to the following formula, (length x width^2^) /2 and was plotted as mean (volume in mm^3^) ± SEM.

### Tumor Chemokine Quantification

Dissected tumor samples were weighed and homogenized in 1 mL cold 1xPBS containing protease inhibitor using a Tissue Tearor 985 370 Handheld homogenizer. The homogenates were centrifuged at 14 000 × g for 15 min to collect supernatants. Chemokines in supernatants were subsequently quantified using LEGENDplex Mouse Proinflammatory Chemokine Panel (BioLegend, 740 368) and analyzed according to the manufacturer instructions. Total protein concentration in each sample was assessed by BCA assay(ThermoFisher Scientific, YC364451) and used to normalize chemokine concentrations to total protein concentration in the supernatant.

### Chemotaxis Assay of CD8^+^ T Cells based on Transwell

Transwell migration of lymphocytes was performed with mature cytotoxic T lymphocytes (CTLs) and concentrated KPC1199‐conditioned medium. In brief, splenic CD8^+^ T cells isolated from OT‐I mice were stimulated with OVA257‐264 for 3 days in the presence of 20 ng mL^−1^ recombinant murine IL‐2 (Novoprotein, CK24). To acquire KPC1199‐conditioned medium, sgCtrL and *Sin3B*sg1 cells were cultured with or without 10 ng mL^−1^ IFNγ for 48 h. The medium was then concentrated (1×, 5×, 10×, or 15×) for chemo‐attractants. In the transwell cell migration assay, 24‐well 5‐µm inserts (Labselect,14 331) were used. Migration medium (600 µL) containing either KPC1199‐conditioned medium with the indicated concentrations or control medium was loaded into the lower chamber. 3×10^5^ splenic CD8^+^ T cells were seeded in 100 µL medium in the upper chamber. After 2 h of incubation at 37 °C under 5% CO2 conditions, the migrated CD8^+^ T cells were collected from the lower chambers and stained with anti‐CD8a antibody (PE‐Cy7, BioLegend, 100 722, 1:200) and DAPI. To normalize samples, beads of known concentration were added to all samples before counting by FACS. The migration ratio was determined by the number of input CD8^+^ T cells. For in *vitro* CXCR3 neutralization, mature cytotoxic CD8^+^ T cells were incubated with AMG487 (10 µM, MCE, HY‐15319) at 37 °C for 1.5 h before adding CD8^+^ T cells to the transwell upper chamber, and then follow the above steps for the experiment.

### Tumor Cell and CD8+ T Cells Co‐Culture Experiment

For examining in vitro T cell proliferation, CD8^+^ T cells isolated from the spleen of C57BL/6J mice were labeled with 10 µM CFSE (eBioscience, 65‐0850‐84) for 10 min at 37 °C in the dark, and washed three times with cold RPMI‐1640 supplemented with 10% FBS. 1×10^4^ PDAC cells were seeded in 96‐well plates one day in advance. Then, CD8^+^ T cells were added in a ratio of 1:5 and 1:10 PDAC cells to T cells and cultured in 200 µL RPMI‐1640 supplemented with 10% FBS, plus 2 µg mL^−1^ anti‐CD3/CD28 (BioLegend, 100 340/102 116) and 20 ng mL^−1^ recombinant murine IL‐2 (Novoprotein, CK24). After 48 h and 72 h, CD8^+^ T cells were harvested and stained with anti‐CD8a antibody (PE‐Cy7 BioLegend, 100 722) and DAPI, and cell proliferation was analyzed by CFSE dilution with flow cytometry.

To measure effector function of CD8^+^ T cells, 1 × 10^4^ PDAC cells were seeded in 96‐well plates one day in advance. Then, isolated splenic CD8^+^ T cells were added in a ratio of 1:1, 1:5, and 1:10 PDAC cells to T cells and co‐cultured for 48 h. Harvested CD8^+^ T cells were stimulated with 1 µg mL^−1^ ionomycin, 100 ng mL^−1^ phorbol 12‐myristate 13‐acetate (PMA) and 5 µg mL^−1^ BFA for 4 h at 37 °C, and then analyzed by intracellular staining of IFN‐γ (anti‐IFN‐γ BV421, BioLegend, 505 830,) and Granzyme B (anti‐Granzyme B PE, BioLegend, 372 208). After removal of CD8^+^ T cells, the viability of remaining PDAC cells was measured using Cell Titer‐Glo (CTG) (Promega, G7572) reagent according to the manufacturer's instructions.

### CRISPR‐Cas9‐Mediated Gene Knockout

Stable *Sin3B* knockout (Sin3B‐KO) and Sin3A‐KO KPC1199 cells were generated by the lentivirus‐mediated CRISPR‐Cas9 technology. The sgRNA oligos for target genes were annealed and cloned into the BsmBI (Thermo Fisher Scientific) digested plasmid lentiCRISPR v2 vector (52 961, Addgene, Cambridge, MA). A non‐targeting sgRNA designated as sgRNA irrelevant was used as control. In brief, pMD2.G, psPAX2 lentiviral packaging plasmids (Addgene) and lentiCRISPR v2 were co‐transfected into 293T cells using polyethylenimine (PEI). After 48 h, lentiviral particles were collected and filtered. Then transduced PDAC cells with lentivirus for 24 h, remove them. All gene‐edited cell lines were validated for knockout efficiency by western blot and/or amplicon sequencing of targeted loci after puromycin (YeaSon, 60210ES60) selection. All sgRNA primers are listed in Table S1 (Supporting Information).

### Gene Knockdown by shRNA

All shRNAs targeting Ms‐*Cxcl9*, Ms‐*Cxcl10*, and human‐SIN3B were purchased from Qinke Inc. (Shanghai, China). Lentiviruses carrying shRNA plasmids were produced by co‐transfecting 293 T cells with two helper plasmids (psPAX2 and pMD2G). After 48 h, the harvested virus supernatant will be harvested and transduced to tumor cells. Transduced cells were selected with GFP sorting or neomycin. Knockdown efficiency was confirmed by western blotting or RT‐PCR. The shRNA oligo sequences for their respective target genes are listed in Table S1 (Supporting Information).

### RNA Extraction and Quantitative Real‐Time PCR

Total RNA was extracted from tumor cell or tissue using TRIzol (MRC, TR118‐200) extraction according to the manufacturer's instructions. RNA was reverse transcribed into cDNA using the High‐Capacity cDNA Reverse Transcript kit (Invitrogen, 4 368 813). The obtained cDNA samples were amplified by RT‐PCR with SYBR Green Mix (Roche,0 491 391 4001). The 2^−ΔΔCT^ method was used to calculate relative gene expression. The results were normalized to the housekeeping gene *Gaphd* and presented as fold change (tested samples over the control). Sequences for qPCR primers are listed in. The primer sequences for target genes are listed Table S2 (Supporting Information).

### Western Blot Analysis and Antibodies

Whole‐cell lysates were prepared using lysis buffer (RIPA lysis buffer, Yeason, 20101ES60) containing PMSF (1 mM) and protease inhibitor cocktail (MedChemExpress, HY‐K0010). The protein concentration of whole‐cell lysates was measured using a BCA Protein Assay kit (ThermoFisher Scientific). The primary antibodies were used included anti‐SIN3B (Novus, NBP2‐20367), anti‐SIN3B (Santa Cruz, sc‐13145 X), anti‐Histone H3 (Abcam, ab1791), anti‐H3K27Ac (Abcam, Ab4729), anti‐H3K27me3 (Abcam, Ab6002), anti‐HDAC1 (Proteintech, 10197‐1‐AP), anti‐HDAC2 (Proteintech, 12922‐3‐AP), anti‐β‐ACTIN (Proteintech, HRP‐60008) and GAPDH (Proteintech, HRP‐60004).

### Luciferase Assay

ISRE reporter and Renilla were co‐transfected in control and *Sin3B*‐KO KPC1199 cells. Transfection was performed using Lipofectamine 3000 Reagent (Thermo Fisher Scientific, L3000015). After 48 h, cells lysis was detected with luciferase assay KIT (Yeason, 11402ES60) according to manufacturer's instructions. The results were normalized to the luciferase activity of Renilla and presented as fold change.

### Immunofluorescence

Tumor tissues from mice were fixed in 10% buffered formalin at 4 °C overnight, paraffin‐embedded and sectioned, and then mounted. Sections were deparaffinized in xylene, rehydrated, and washed in PBS. Subsequently, sections were boiled for 15 min with antigen unmasking solution (MXB, MVS‐0101) and cooled naturally to room temperature, blocked in PBST with 5% goat serum for 1 h at room temperature, and incubated with primary antibodies against CK19 (DSHB, TROMA‐III) and CD8a (Invitrogen, 14‐0808‐82) in PBST with 1% goat serum at 4 °C overnight in a humidified box. The next day, the slides were incubated with Alexa Fluor 488‐conjugated secondary antibody (Invitrogen, A11008), or Alexa Fluor 594‐conjugated secondary antibody (Invitrogen, A11005) in PBS for 1hr at room temperature. Cell nuclei were counterstained with DAPI (BioLegend, 422 801). Fluorescence images were acquired using a fluorescence microscope.

### Flow Cytometry

To characterize the subpopulations of the tumor immune microenvironment, orthotopic tumors were obtained at the end of study. Tumors were isolated and minced into small pieces and digested with 2 mg mL^−1^ collagenase IV (Yeason, 40510ES60) at 37 °C for 25 min. Dissociated tumor cells were filtered through a 100 µm filter to achieve a single‐cell suspension before staining for flow cytometry analysis. For cell surface staining, single‐cell suspensions were incubated with antibodies diluted in staining buffer (2% FBS in PBS) at 4 °C in the dark for 30 min. For intracellular cytokine staining, immediately after isolation, cells were cultured in RPMI medium with 10% FBS and stimulated with PMA (100 ng mL^−1^; Yeasen, 50601ES03), ionomycin (1 µg mL^−1^; Yeasen, 50401ES03) and brefeldin A (5 µg mL^−1^; BioLegend, 420 601) at 37 °C for 4 h. Cells were processed for surface marker staining as described above and then fixed and permeabilized using a Foxp3 Transcription Factor Staining Buffer Set (Invitrogen, 2 271 994) followed by cytokine antibody incubation for 30 min at 4 °C. Antibodies were from BioLegend indicated, anti‐CD45.2‐APCcy7/APC/AF700 (109 824;109 814; 109 822), EpCAM‐PEcy7 (118 216), anti‐CD3‐APC‐Cy7 (100 221), anti‐CD8‐PEcy7 (100 722), anti‐CD4‐BV650 (100 469), anti‐NK1.1‐APC/APCcy7(108 710;108 723), anti‐TCRγ/δ‐PE/APC/AF488(118 108;118 115;107 512), CD19‐FITC(115 505), anti‐CD11b‐Percp‐Cy5.5(101 228), CD11c‐APCcy7 (117 324), anti‐F4/80‐BV421 (123 132), anti‐Ly6G PE (127 607), anti‐Ly6C‐PB (128 014), LAG3‐AF488(369 325), anti‐CD206‐FITC (141 704), anti‐PD‐1 FITC (135 214), anti‐Granzyme B‐PE (372 208), anti‐TNF‐a PE (506 305); anti‐TIM3‐Percp‐Cy5.5 (134 012), anti‐CD44‐PB/FITC (103 020;103 005), anti‐Ki67 FITC/PE (652 417;652 403), MHCII (I‐A/I‐E)‐AF700 (107 622), CXCR3‐PE (155 904), PD‐L1‐BV650(124 336). iNOS‐FITC (2 264 918) was bought from Invitrogen. Data were acquired using a LSRFortessa (BD Bioscience) and analyzed using FlowJo software.

### Immunoblotting and Co‐Immunoprecipitation (Co‐IP)

Cells were lysed with RIPA lysis buffer (Yeason, 20101ES60) with freshly added phosphatase and protease inhibitors. Centrifuge the lysate at 12 000 rpm in a precooled centrifuge for 15 minutes, 4 °C; Immediately transfer the supernatant to a fresh centrifuge tube and discard the pellet. The supernatant was pre‐cleaned with protein A or protein G Sepharose beads (Thermo Scientific, 20 421) for 60 min at 4 °C, 10% of the supernatant was stored as input, and the remainder was incubated with 4 µg of antibody and 50 µL of protein A or protein G Sepharose beads overnight at 4 °C. The immunocomplexes were then washed with IP buffer (Beyotime, P0013) and separated by SDS‐PAGE Gel. Immunoblotting was performed following standard procedures.

### Mass Spectrometry Analysis

Whole‐cell lysates were prepared using RIPA lysis buffer containing PMSF and protease inhibitor cocktail and were centrifuged at 12 000 g for 10 min. The obtained supernatants were incubated with 5 µg anti‐SIN3B antibody from Santa Cruz (sc‐13145 X) or Novus (NBP2‐20367) and 50 µL of protein A or protein G Sepharose beads overnight at 4 °C. The immunocomplexes were then washed with IP buffer and separated by SDS‐PAGE Gel. The SDS‐PAGE Gel containing proteins was digested by Trypsin buffer (Promega, V5113) for Mass Spectrometry analysis (MS). LC‐MS/MS analysis was performed on mass spectrometer (Thermo Fisher Scientific; Orbitrap Fusion Lumos) that was coupled to HPLC (Thermo Fisher Scientific). The MS data were analyzed and searched against the database (Mus_musculus. GRCm39) using Proteome Discover software (version 2.4). The search followed an enzymatic cleavage rule of Trypsin/P and allowed maximal two missed cleavage sites and a mass tolerance of 10 ppm for fragment ions. Carbamidomethylation of cysteines was defined as fixed modification, while protein N‐terminal acetylation and methionine oxidation were defined as variable modifications for database searching. The cutoff of global false discovery rate (FDR) for peptide and protein identification was set to 0.01. Protein abundance was calculated on the basis of the normalized spectral protein intensity (LFQ intensity).

### RNA‐Seq Analysis

RNA‐seq reads were aligned to the Mouse‐mm9 (KPC1199) genomes by HISAT2 with default parameters. Read counts were calculated by feature counts using uniquely mapped reads, and differentially expressed genes were identified by DESeq2 with a cut‐off of more than 1.5 or 1.3‐fold‐change and a *P* value of less than 0.05. To identify DEGs (differential expression genes) between two different samples, the expression level of each transcript was calculated according to the transcripts per million reads (TPM) method. DEGs of RNA‐seq are list in Table S3 (Supporting Information).

### CUT&Tag Analysis

The CUT&Tag assay was performed following the manual of the hyperactive pG‐Tn5/pA‐Tn5 transposase for CUT&Tag kit (Vazyme, TD901) as described. DNA libraries were prepared for the Trueprep index kit v2 (Vazyme, TD202) according to the manufacturer's instructions. Anti‐H3K27ac (Abcam, ab4729, 0.5  µg per sample), anti‐SIN3B (Novus, NBP2‐20367, 1 µg/sample) and anti‐HDAC1(Proteintech, 10197‐1‐AP, 1 µg per sample) were used. Reads were trimmed of adaptor sequences using Fastp (v 0.20.1) and aligned to the mm10 mouse genome using Bowtie2 (v 2.4.2) with the following parameters, ‐very‐sensitive‐local‐no‐unal‐no‐mixed‐no‐discordant‐phred33 ‐I 10 ‐X 700. Reads were then normalized by calculating the reads per kilobase of transcript per million mapped reads (RPKM). Peak calling used macs2 (v 2.2.7.1) with the following parameters, ‐B ‐t ‐broad ‐g mm ‐broad‐cutoff 0.05. Bigwig files were generated using deepTools (v.3.1.3). Heatmaps normalized to RPKM were generated using the deep Tools function plot Heatmap or using the R package ComplexHeatmap (v.2.4.3). Figures illustrating these continuous tag counts over selected genomic intervals were created using the IGV browser. To define Sin3B‐binding sites, the lost Sin3B peaks in *Sin3B*‐KO cells as compared to control cells was calculated with a sliding window of 6 kb across the whole genome. To define H3K27Ac peaks regulated by Sin3B, all H3K27Ac peaks located in the Sin3B‐binding sites were selected and shown (Figure 5H). For signal correlation, reads that fell within consensus SIN3B peaks annotated loci were first counted using the annotatePeaks.pl script with the Homer package for CUT&Tag markers, and log_10_ signal was calculated for each region with R. Signal correlation was determined using two‐tailed Pearson tests.

### TF Motif Enrichment

Motif enrichment analysis was performed individually on the overlap genes(Figure 5J) using the HOMER de novo motif discovery tool using find Motifs Genome command with size  = given and length  =  8,10,12 parameters.

### Human PDAC Cohorts

*Tissue Microarray of Surgical PDAC Samples (Renji Cohort 1)*: 121 samples were used derived from patients diagnosed with pancreatic ductal adenocarcinoma at Renji Hospital in Shanghai, China, spanning the period from 2017 to 2018. All patient tissue samples were collected with informed consent specifically for research purposes, following approval by the Local Ethics Committee. The samples were obtained from formalin‐fixed paraffin‐embedded (FFPE) tissues of PDAC patients; a pathologist examined FFPE tissue sections from each case and selected representative regions of invasive tumor for coring; two punches from each specimen were obtained and embedded into a master tissue microarray (TMA) block. TMA slides were processed as previously described. In brief, TMA slides were deparaffinized and hydrated followed by antigen retrieval. Slides were boiled for 15 min in citrate buffer (MXB, MVS‐0101) and cooled naturally to room temperature for antigen retrieval. Subsequently, slides were blocked in PBST with 5% goat serum for 1 h at room temperature, and incubated with anti‐SIN3B (Novus, NBP2‐20367, 1:100), and anti‐CD8a (Invitrogen, 14‐0808‐82, 1:50) respectively in PBST with 1% goat serum at 4 °C overnight in a humidified box. The next day, the slides were incubated with corresponding secondary antibodies goat anti‐Rat IgG (H+L) (Invitrogen, 31 470, 1:200)and goat anti‐Rabbit 3 IgG (H+L) (Sigma, AP132P, 1;200). Patient information is comprehensively detailed in Table S5 (Supporting Information).

*Human PDAC Patients with Chemotherapy Plus Anti‐PD1 Treatment (Renji Cohort 2)*: 61 unselected metastatic PDAC were selected from the PDAC database treated between Jan 2020 and Dec 2021 in Renji hospital, School of Medicine, Shanghai Jiao Tong University. Ethical approval for this retrospective study was obtained under reference KY2020‐116. Patients with metastatic PDAC receiving anti‐PD1 antibodies in combination with chemotherapy were enrolled in this study. These patients first received six cycles of chemotherapy combined with PD‐1 antibody and then received PD‐1 antibody alone as maintenance therapy. The response to the therapy was evaluated according to the criteria of Response Evaluation Criteria in Solid Tumors (RECIST1.1). In the cohort, 21 were surgically resected PDAC specimens and 40 were biopsy specimens. Tissues were fixed in 10% neutral buffered formalin for 48 h and embedded in paraffin. All patient tissue samples were collected with informed consent for research use and local ethics committee approval. Treatment information is listed in Table S6 (Supporting Information).

### Statistical Analysis

Statistical analyses were performed using GraphPad Software (Prism 9.0). All data are presented as the mean ± SD (in vitro), ± SEM (in vivo) or mean with range. Unless otherwise stated, one‐way or two‐way ANOVA together Tukey's multiple comparisons test were used to determine statistical significance among multiple groups. Unpaired Student's t test was used to determine statistical significance between two groups. Unless indicated, the results are from at least two or three independent experiments.

## Conflict of Interest

The authors declare no conflict of interest.

## Author Contributions

Z.Z., Y.T., and Y.W. contributed equally to this work. ZY.Z. and J.X. designed the experiments and interpreted the data. ZY.Z. and YY.T. performed most of the experiments. ZY.Z. and YY.T. performed the bioinformatics analysis under the guidance of J.X. and YJ.T. Y.W performed the retrospective study. M.M. and YW.S. provided key materials. JY.X., XT.Y., MZ.L. and NN.N., assisted in some experiments. ZY.Z. and J.X. wrote the manuscript. J.X. provided overall guidance.

## Supporting information



Supporting Information

Supplemental Table 1

Supplemental Table 2

Supplemental Table 3

Supplemental Table 4

Supplemental Table 5

Supplemental Table 6

## Data Availability

All genomic sequencing data that involved in this study have been deposited in the Gene Expression Omnibus database with the accession code GSE254767 and GSE254770.
