# Supplementary material for: SIN3B Loss Heats up Cold Tumor Microenvironment to Boost Immunotherapy in Pancreatic Cancer
Source: Adv Sci (Weinh). 2024 Sep 24;11(43):2402244. doi: 10.1002/advs.202402244 (PMC11578377; doi:10.1002/advs.202402244)
Supplement: Supplementary file 1 — Supporting Information [file ADVS-11-2402244-s002.docx]

**Supplementary Figures**

**
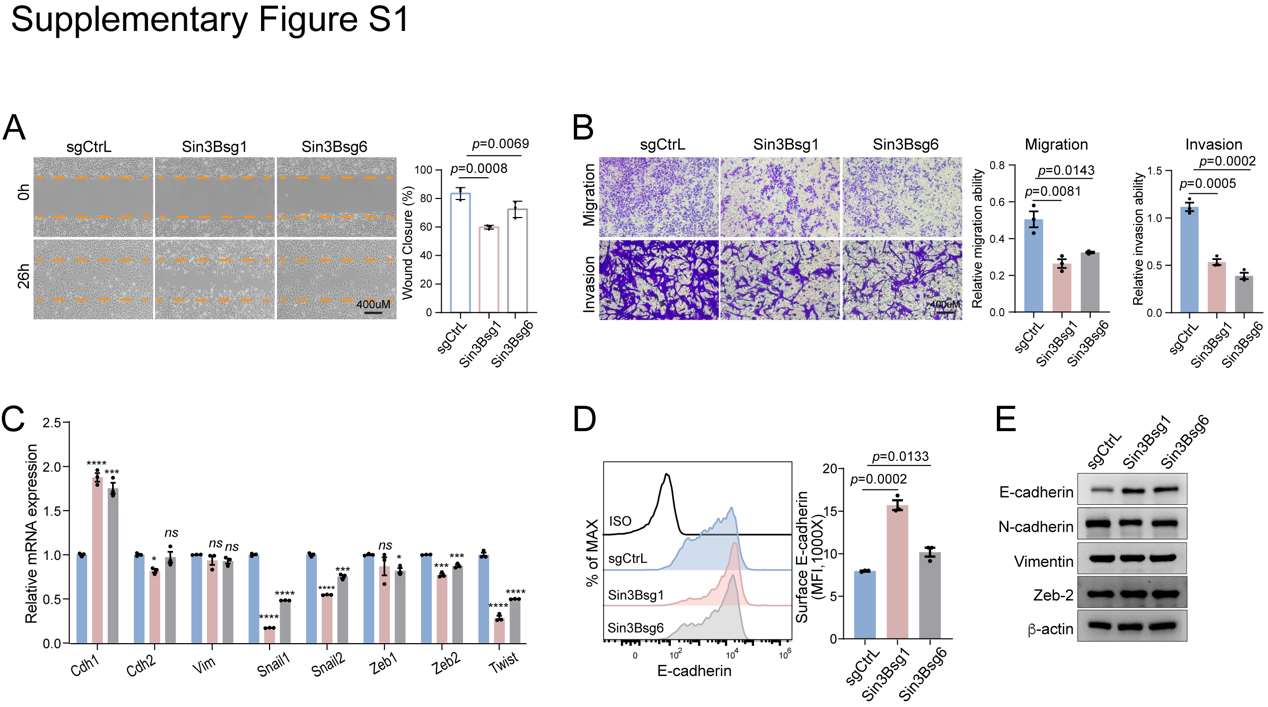
**

**Supplementary Figure S1**

(A) Wound healing assay of sgCtrL, Sin3Bsg1, and Sin3Bsg6 KPC1199 cells after 26 hours. Representative images were captured using phase contrast microscopy. Scale bars=400 µm.

(B) Transwell-based migration and invasion assay of sgCtrL, Sin3Bsg1, and Sin3Bsg6 KPC1199 cells over 24 hours. Relative migration and invasion capabilities were quantified with OD595. Scale bars=400 µm.

(C) RT-PCR analysis quantifying the expression levels of epithelial-mesenchymal transition (EMT)-related genes in control and Sin3B-deficient KPC1199 cells.

(D) Representative histograms (left) and statistical analysis (right) of E-cadherin in control and Sin3B-deficient KPC1199 cells

(E) Western blot analysis of indicated proteins in KPC1199 cells.

All data are presented as the mean ± SEM. One-way ANOVA with multiple comparisons in A-D. ns, not significant.

**
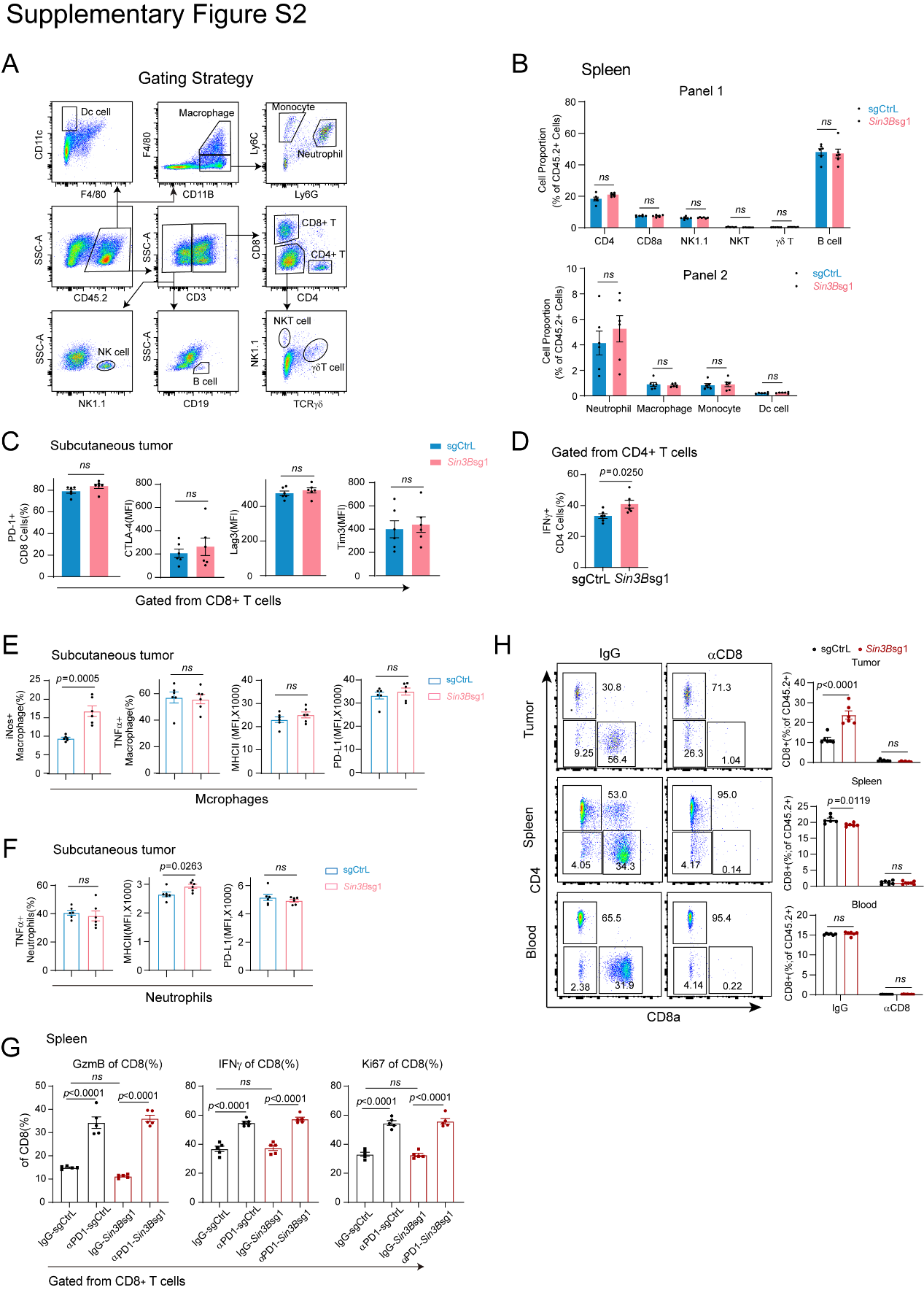
**

**Supplementary Figure S2**

(A) Gating strategy for flow cytometry analysis of tumors and spleens used in this study.

(B) Composition of key immune cells in the spleens of C57BL/6J wildtype mice inoculated with either sgCtrl or Sin3Bsg1 KPC1199 cells.

(C) Statistical analysis of exhaustion markers, PD1, CTLA4, Lag3, and Tim3 production in tumor-infiltrating CD8^+^ T cells from the indicated mice.

(D) Statistical analysis of IFN𝛾 production in tumor-infiltrating CD4^+^ T cells from the indicated mice.

(E) Quantification of iNOS, TNFα, MHCII, and PD-L1 within tumor-infiltrating macrophages from the indicated mice.

(F) Quantification of TNFα, MHCII, and PD-L1 within tumor-infiltrating neutrophils from the indicated mice.

(G) Quantification of GzmB, IFN𝛾, and Ki67 in CD8^+^ T cells from the spleens of the indicated mice.

(H) Blocking efficiency of CD8^+^ T cells in subcutaneous models.

All data are presented as the mean ± SEM. n=5-6 for each group. Unpaired Student’s t-test applied in B-H. One-way ANOVA with multiple comparisons in G. ns, not significant.

**
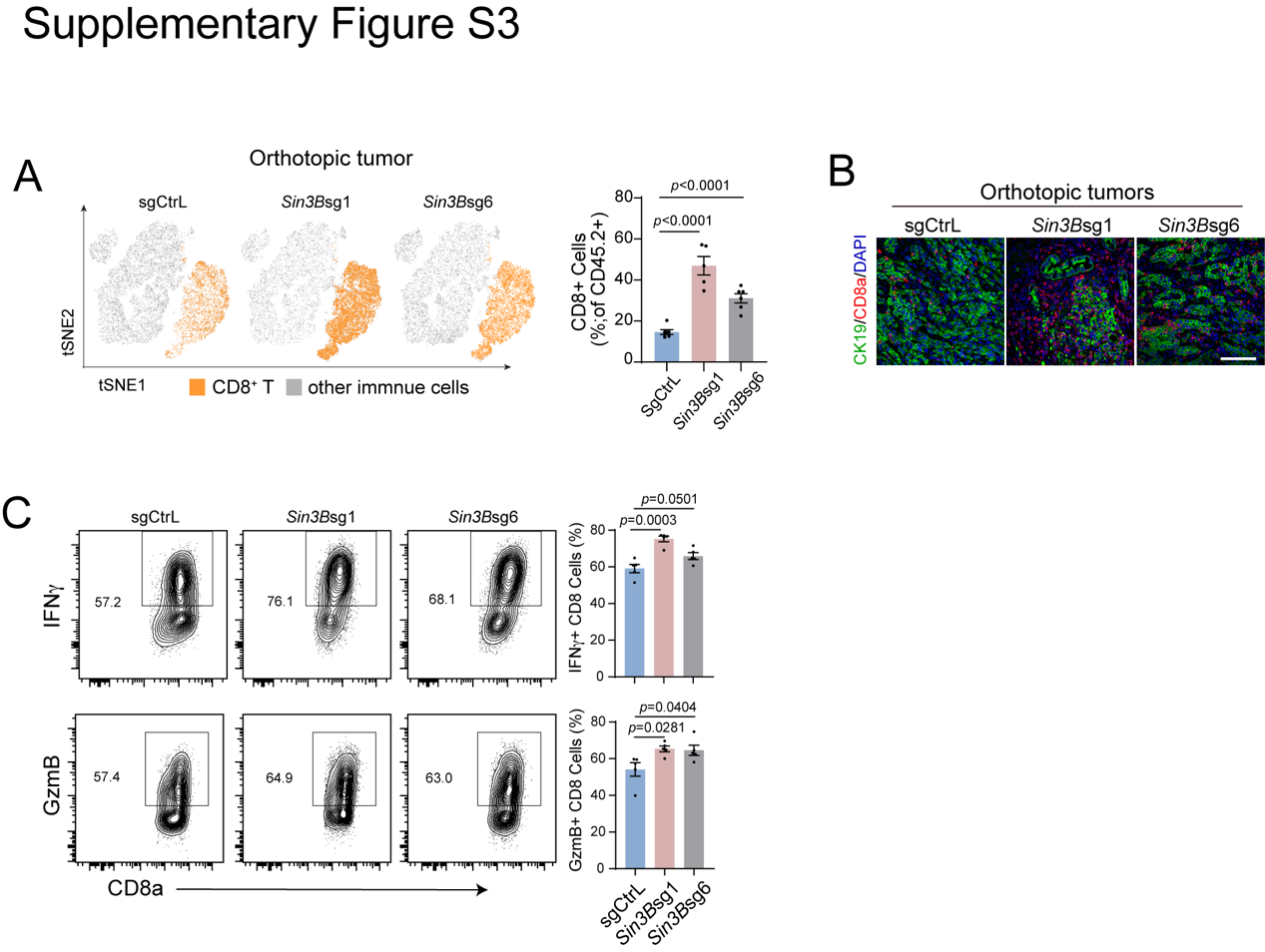
**

**Supplementary Figure S3**

Tumor Immune Microenvironment in Orthotopic Tumors

(A) t-SNE plots illustrating the composition of CD8+ T cells in control and Sin3B deficiency orthotopic tumors from C57BL/6J mouse models.

(B) Immunofluorescence staining for tumor cells (CK19) and CD8+ T cells (CD8a) in orthotopic tumors. Scale bar =40X.

(C) Representative contour plots (left) and statistical analysis (right) of GzmB and IFN𝛾 production by tumor-infiltrating CD8+ T cells from the specified mice.

All data are presented as the mean ± SEM. n=5 for each group. One-way ANOVA with multiple comparisons was used in A and C. ns, not significant.

**
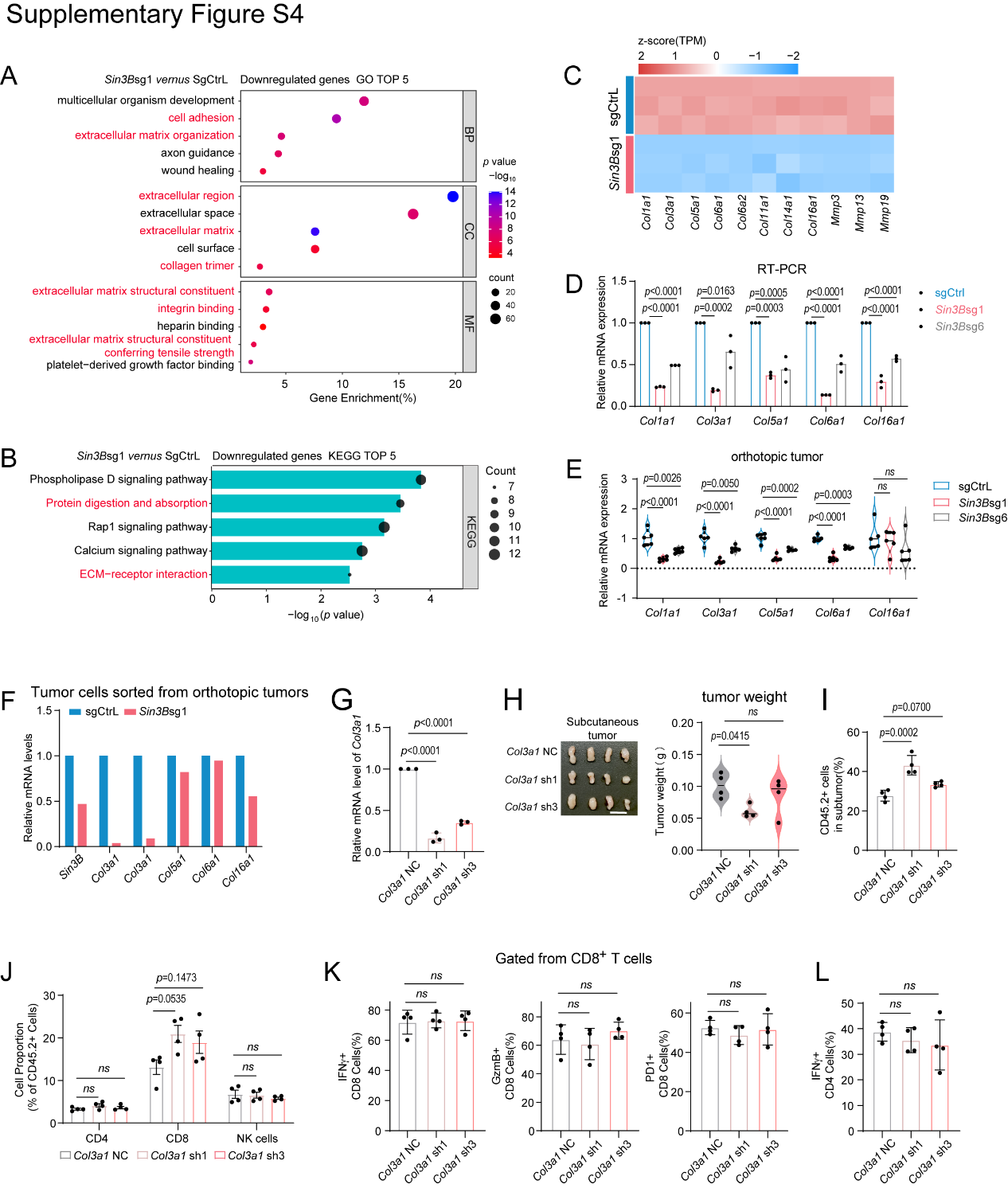
**

**Supplementary Figure S4**

Minor contribution of downregulated Col3a1 to TIME remodeling upon Sin3B loss

(A and B) Gene ontology (GO) analysis (A) and KEGG pathway analysis (B) of RNA-seq data, highlighting the top 5 enriched pathways downregulated in Sin3B-deficienct KPC1199 cells compared to sgCtrl cells. Categories include Biological Process (BP), Cellular Component (CC), and Molecular Function (MF).

(C) Heatmap representation of the expression levels of ECM-associated genes (identified in A) in control and Sin3B deficiency PDAC cells, as determined by RNA-seq data.

(D and E) Expression levels of selected collagen genes (Cols) in PDAC cell lines cultured in vitro (D) and in bulk tissue from orthotopic tumors (E) were quantified using RT-PCR. For E, n=5 for each group.

(F) Expression levels of selected Cols in purified PDAC tumor cells isolated from orthotopic tumors were determined using RT-PCR.

(G) PDAC cells (KPC1199) subjected to Col3a1 knockdown (KD) via shRNA, with the efficiency of this knockdown confirmed by RT-PCR. NC represents the negative control.

(H) Presentation of subcutaneous tumors and tumor weight in C57BL/6J wildtype mice injected with Col3a1 NC or KD (Col3a1 sh1, Col3a1 sh3) KPC1199 cells (n=4 for each group).

(I) Quantification of the immune cell (CD45.2+) percentage in subcutaneous tumors from the specified mice, as assessed by flow cytometry.

(J) Analysis of the composition of T cells and NK cells in subcutaneously implanted tumors with either NC or cCol3a1 sh.

(K) Statistical evaluation of GzmB and IFNγ production by tumor-infiltrating CD8+ T cells, and assessment of PD-1 surface expression on tumor-infiltrating CD8+ T cells.

(L) Statistical evaluation of IFNγ production by tumor-infiltrating CD4+ T cells.

Data are presented as the mean ± SEM unless otherwise indicated. For H-L, and n=4 for each group. One-way ANOVA with multiple comparisons in D-L except F. ns, not significant.

**
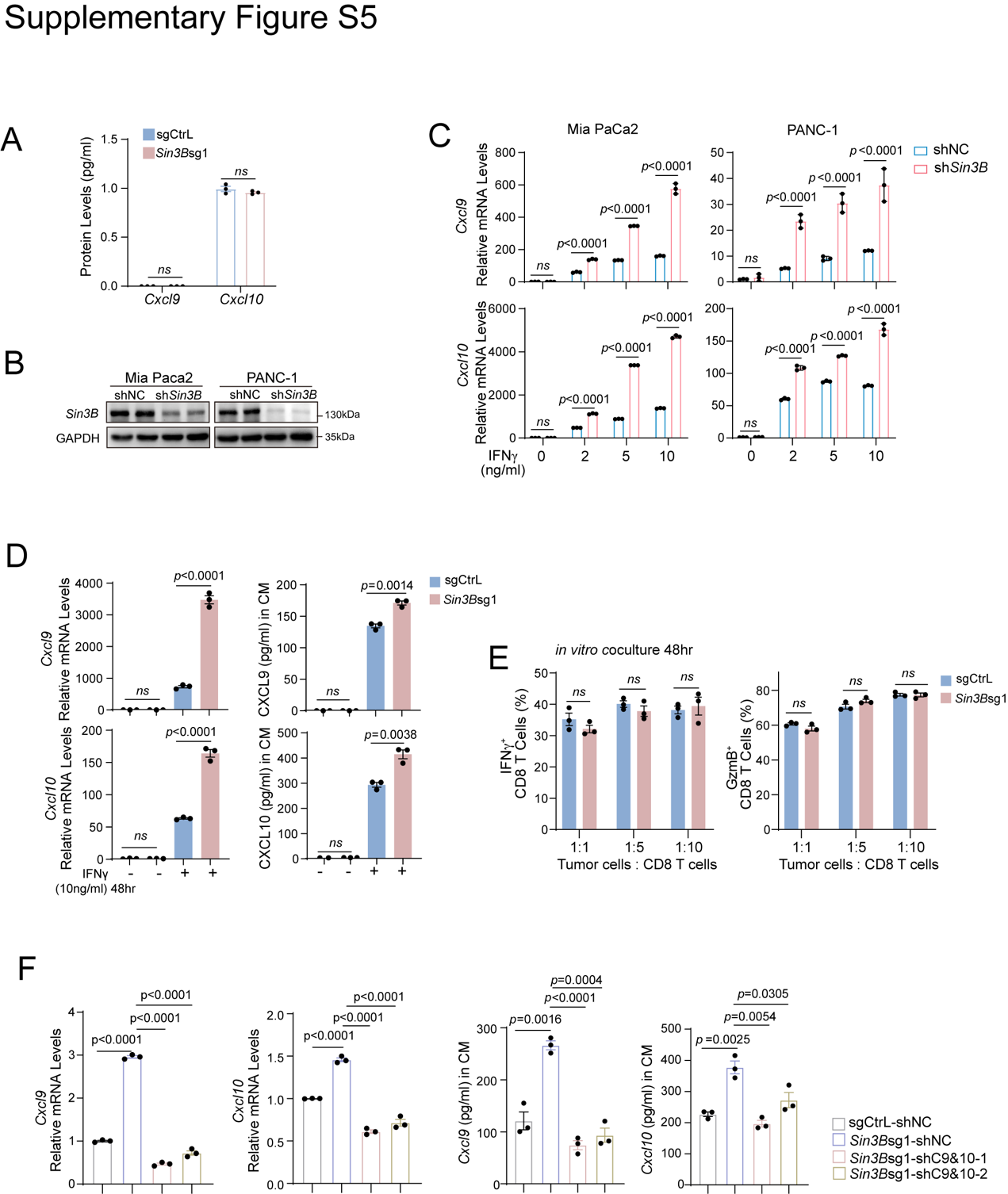
**

**Supplementary Figure S5**

(A) The protein levels of *Cxcl9* and *Cxcl10* in the CM derived from KPC1199 were measured.

(B) Human PDAC cells, Mia Paca2 and PANC-1, with *Sin3B* knockdown were constructed using shRNA, and the efficiency of this knockdown was validated through western blot analysis. NC, negative control.

(C) RT-PCR analysis quantified *Cxcl9* and *Cxcl10* mRNA expression levels in human PDAC cells after treatment with varying IFNγ concentrations for 24 hours.

(D) mRNA expression (left) and the protein levels (right) of *Cxcl9* and *Cxcl10* in KPC1199 cells, following treatment with 10ng/ml IFNγ for 48 hours.

(E) Co-culture of sorted CD8^+^ T cells and control or *Sin3B*-deficienct KPC1199 cells for 48h *in vitro*, tumor cell: CD8=1:1, 1:5,1:10. Quantification of IFNγ and GzmB in CD8^+^ T cells.

(F) *Sin3B*-deficienct KPC1199 cells with *Cxcl9* and *Cxcl10* knockdown were constructed using shRNA, and the efficiency of this knockdown was validated through mRNA and protein levels of *Cxcl9* and *Cxcl10*. NC, negative control. sgCtrL-shNC, Sin3Bsg1-shNC, Sin3Bsg1-shCXCL9&CXCL10-1 (Sin3Bsg1-shC9&10-1), Sin3Bsg1-shCXCL9&CXCL10-2 (Sin3Bsg1-shC9&10-2).

All data are presented as the mean ± SEM. Unpaired t-test in A-E. One-way ANOVA with multiple comparisons in F. ns, not significant.


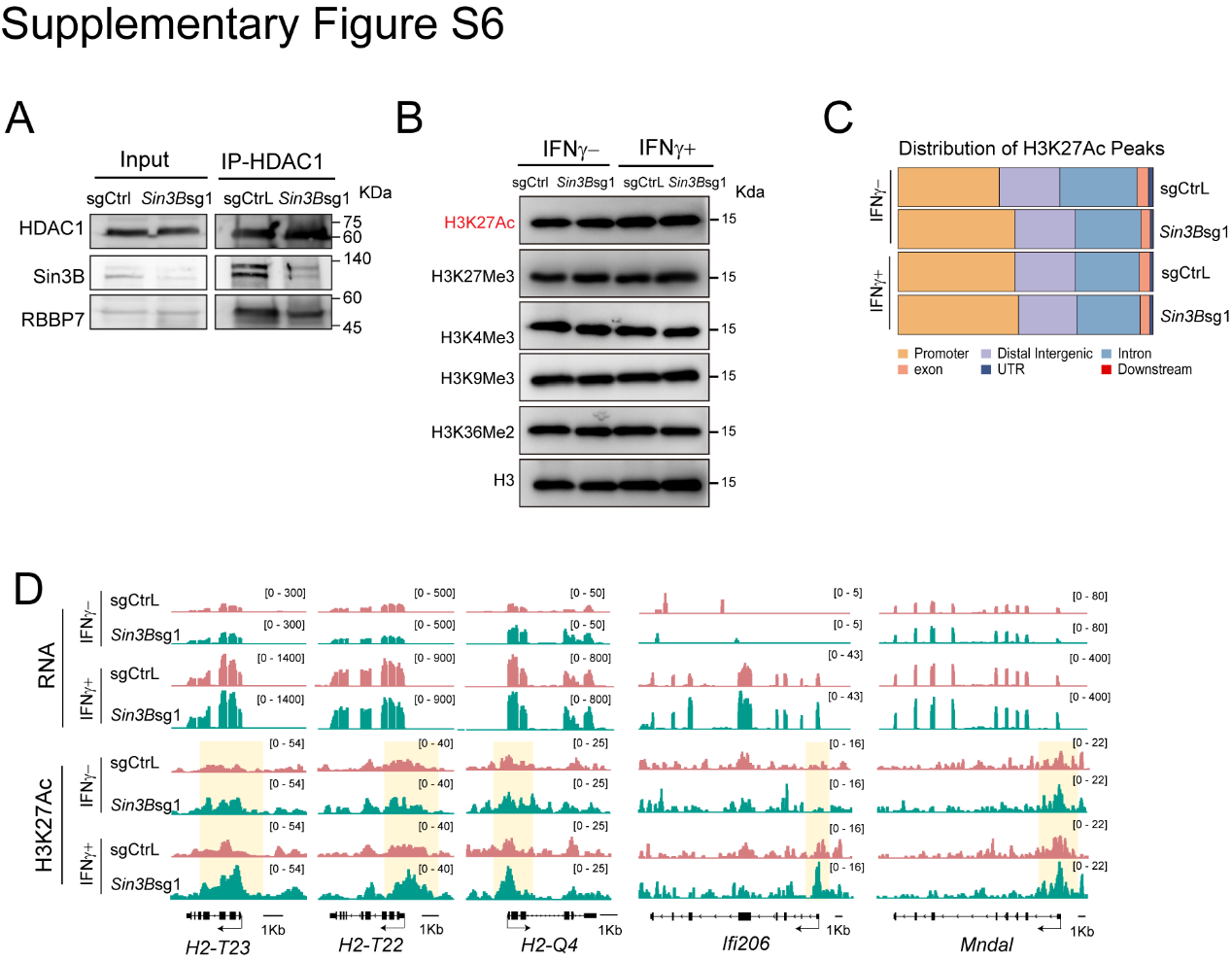


**Supplementary Figure S6**

(A) Co-immunoprecipitations with anti-HDAC1 antibody in control and Sin3B-deficienct KPC1199 cells. Immunoblots were performed using the indicated antibodies. 4% input. IP, immunoprecipitation.

(B) Western blot analysis of indicated epigenetic factors in KPC1199 cells.

(C)Genomic annotations of H3K27Ac binding peaks from H3K27Ac CUT&Tag in control and Sin3B-deficienct KPC1199 cells.

(D)IGV plots showed the RNAseq peaks (upper panel) and Cut&tag peaks of H3K27Ac (lower panel) at the other ISG sites in control and Sin3B-deficienct KPC1199 cells.


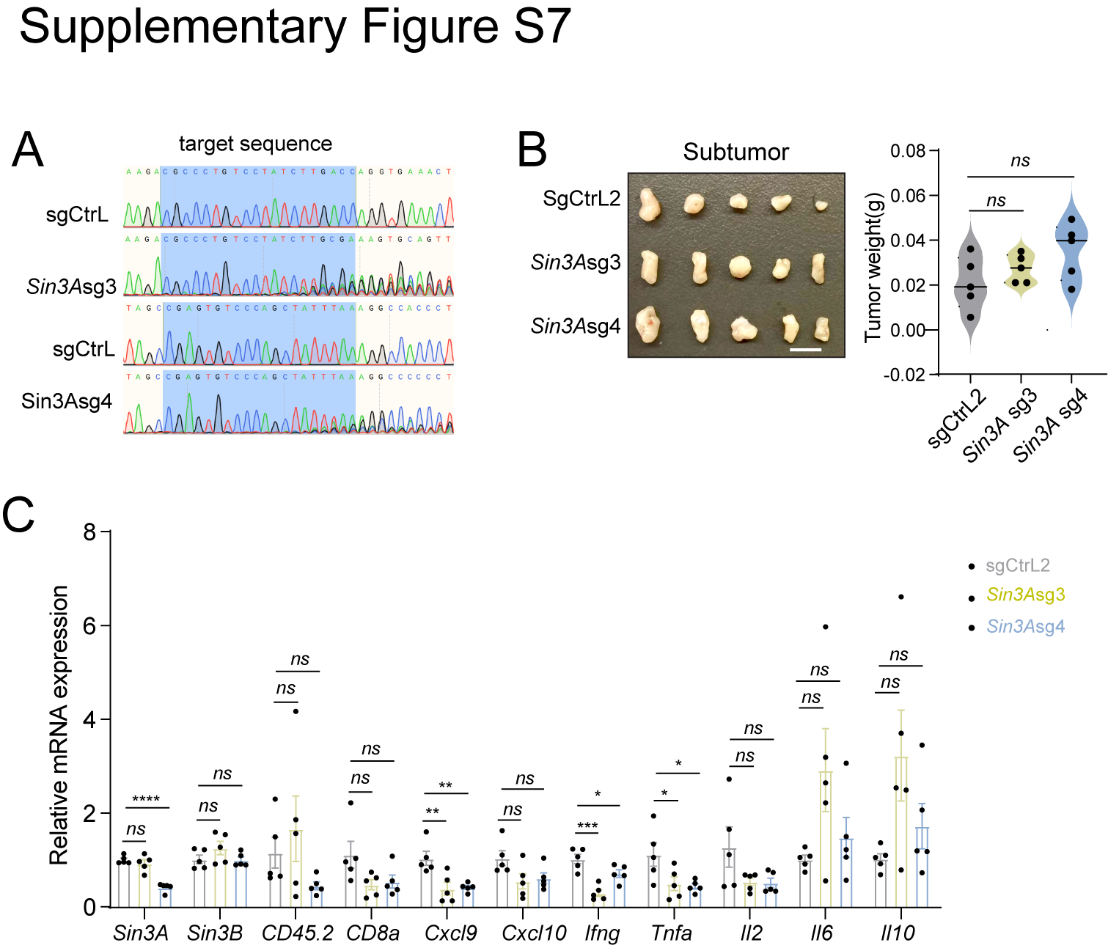


**Supplementary Figure S7**

(A) Using the CRISPR-Cas9 system, *Sin3A* knockout (KO) KPC1199 cells were engineered. The peak chart depicts the DNA sequence of Sin3A specifically targeted by sgRNAs.

(B) Tumor representation and weight were determined for wild-type C57BL/6J mice inoculated with either sgCtrL2 or *Sin3A*-deficienct (Sin3Asg3, Sin3Asg4) KPC1199 cells (n=5 for each group).

(C) The expression levels of *Sin3A*, *Sin3B*, immune cell marker, chosen chemokines and cytokines in subcutaneous tumors were quantified utilizing RT-PCR.

Data are presented as the mean ± SEM. One-way ANOVA with multiple comparisons applied in B and C. ns, not significant, **p*<0.05, ***p*<0.01, ****p*<0.001, *****p*<0.0001.


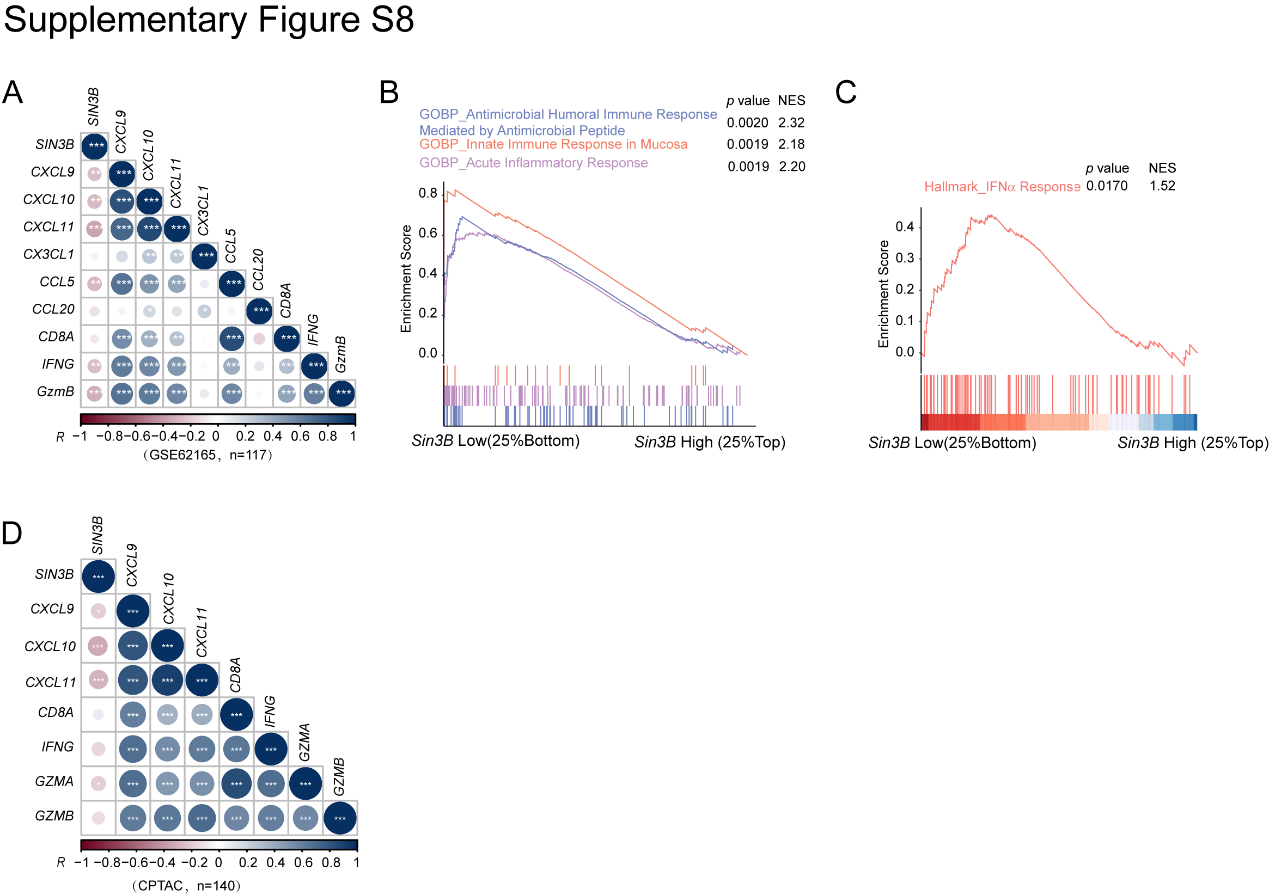


**Supplementary Figure S8**

(A-C) Investigating the role of Sin3B in human PDAC based on the analysis of human PDAC cohort (GSE62165, n=117).

(A) Pearson’s correlation analysis was conducted to assess the relationship between SIN3B expression and the levels of CXCL9, CXCL10, CXCL11, CCL5, CCL20, CD8A, IFNG and GZMB in human primary PDAC transcriptomic data (n=117 samples).

(B-C) GSEA was performed comparing lower quartile Sin3B expressers (Sin3B Low; 25% Bottom) with the upper quartile expressers (Sin3B High; 25% Top) within the human PDAC cohort.

(D) Pearson’s correlation analysis was conducted to assess the relationship between SIN3B expression and the levels of CXCL9, CXCL10, CXCL11, CD8A, IFNG, GZMA and GZMB in human primary PDAC transcriptomic data (CPTAC-data, n=140 samples).

All data are presented as mean ±SEM. **p*<0.05, ***p*<0.01, ****p*<0.001, *****p*<0.0001.


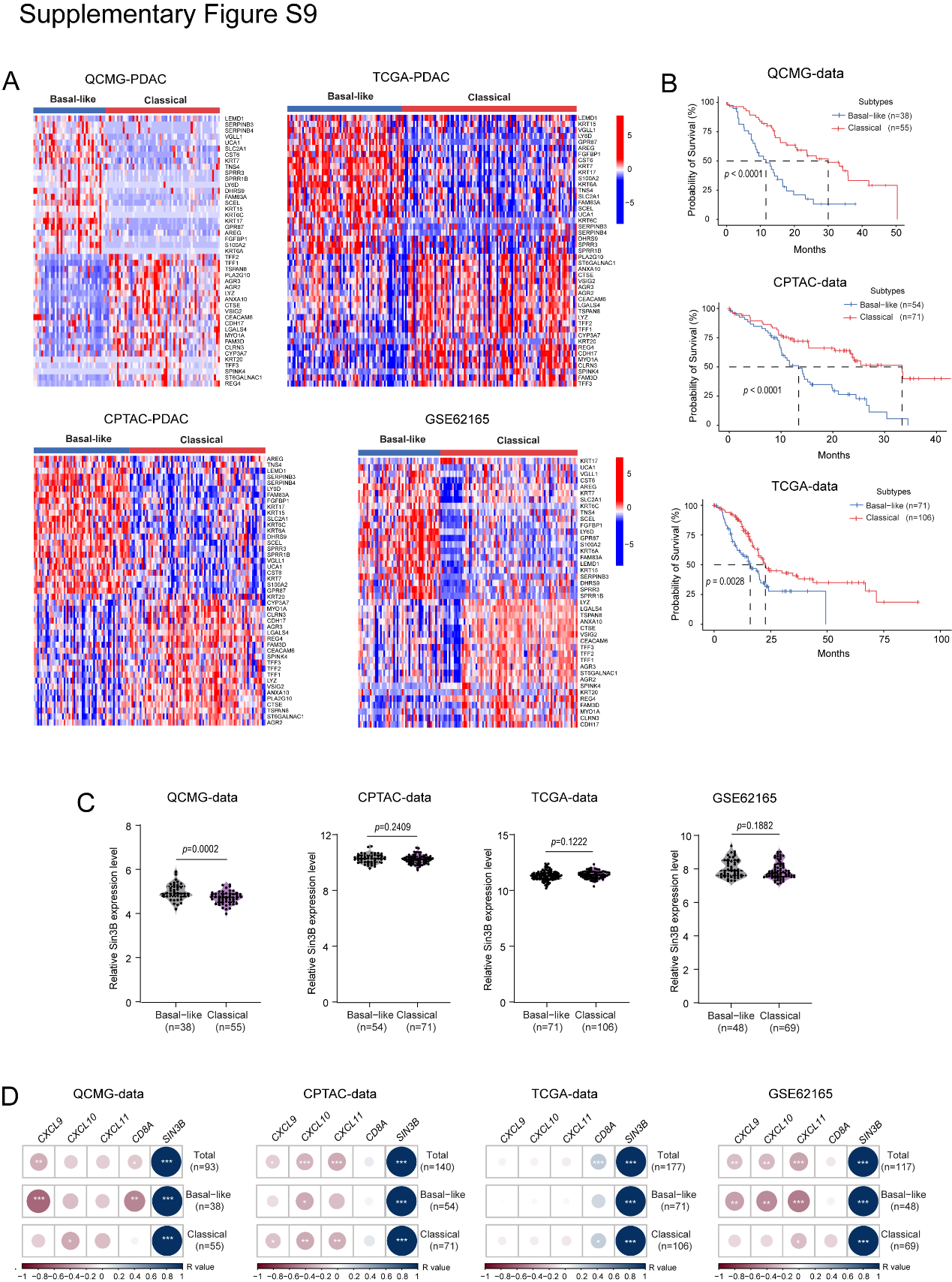


**Supplementary Figure S9**

A-D are derived from the QCMG, CPTAC, TCGA, and GSE62165 PDAC transcriptomics databases. (A) Classification of PDAC into basal-like and classical subtypes based on NMF clustering analysis. (B) Prognostic analysis of basal-like and classical subtypes in PDAC. (C) Expression levels of SIN3B in basal-like and classical subtypes. (D) Pearson’s correlation analysis evaluating the relationship between SIN3B expression and the levels of CXCL9, CXCL10, and CXCL11 in human PDAC transcriptomic data. All data are presented as mean ±SEM. **p*<0.05, ***p*<0.01, ****p*<0.001.

**
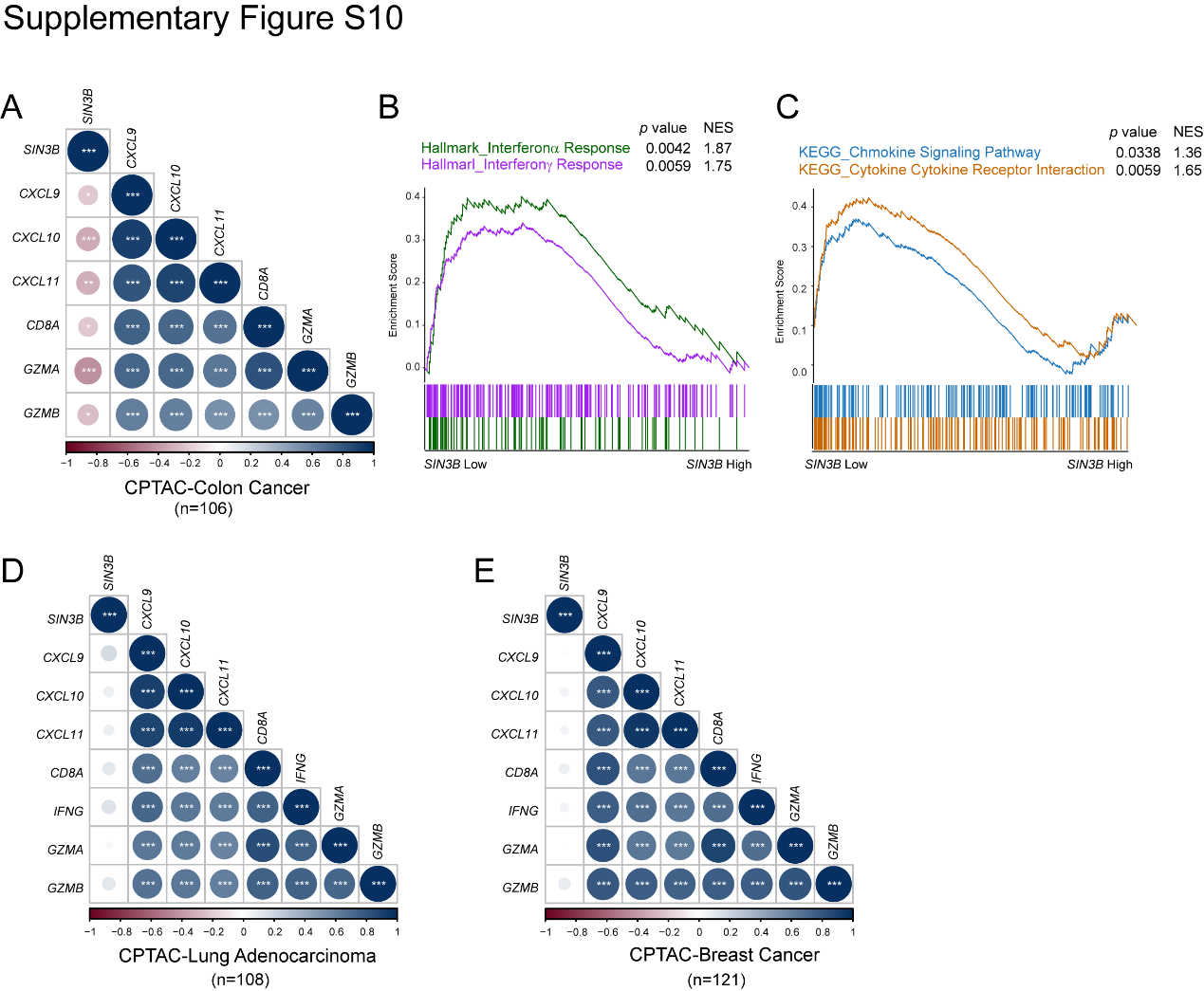
**

**Supplementary Figure S10**

(A-C) Investigating the role of SIN3B in human colorectal cancer (CRC) based on the analysis of human cohort (CPTAC-Colon Cancer, n=106). (A) Pearson’s correlation analysis was conducted to assess the relationship between SIN3B expression and the levels of CXCL9, CXCL10, CXCL11, CD8A, GZMA and GZMB in human CRC transcriptomic data (n=106 samples). (B-C) GSEA was performed comparing lower quartile SIN3B expressers (SIN3B Low; 25% Bottom) with the upper quartile expressers (SIN3B High; 25% Top) within the human CRC cohort.

(D and E) Pearson’s correlation analysis was conducted to assess the relationship between SIN3B expression and the levels of CXCL9, CXCL10, CXCL11, CD8A, IFNg, GZMA and GZMB in human Lung Adenocarcinoma (n=108 samples) and breast cancer transcriptomic data (n=121 samples). **p*<0.05, ***p*<0.01, ****p*<0.001.
